# Supplementary material for: Synthesis of Mono- and Polyazole Hybrids Based on Polyfluoroflavones
Source: Molecules. 2023 Jan 15;28(2):869. doi: 10.3390/molecules28020869 (PMC9865898; doi:10.3390/molecules28020869)

molecules

MDPI

Figure S1. ^{1}H NMR spectrum of compound **4**

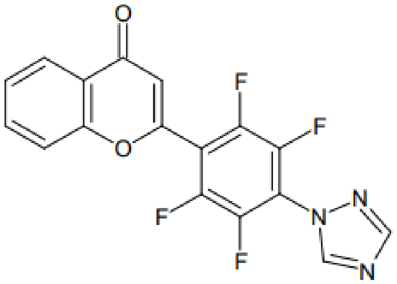

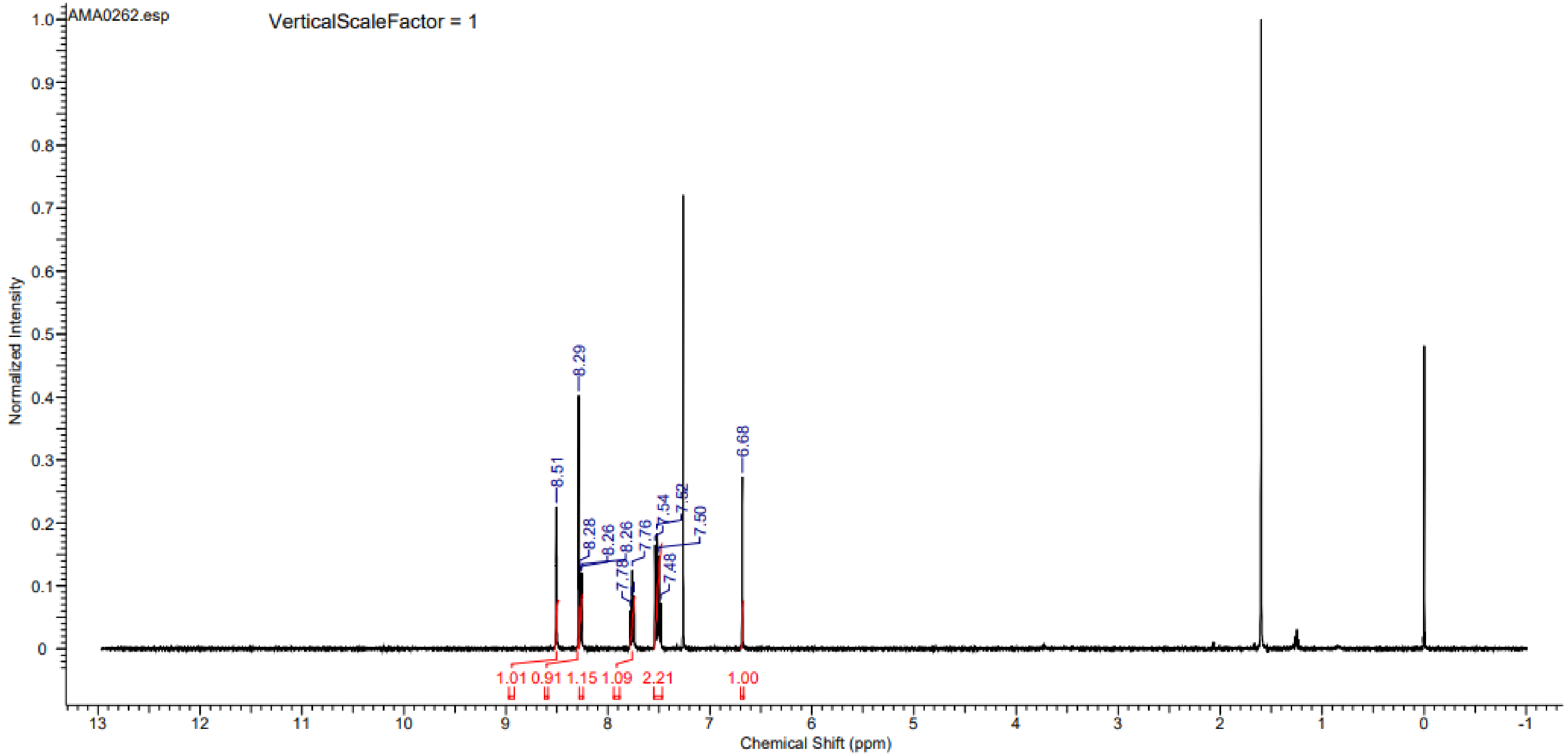

Figure S2. ^{13}C NMR spectrum of compound **4**

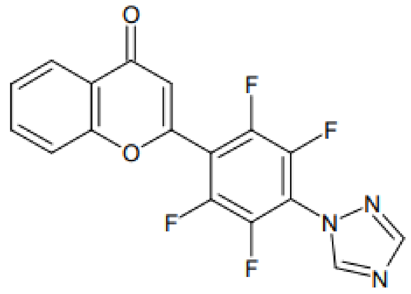

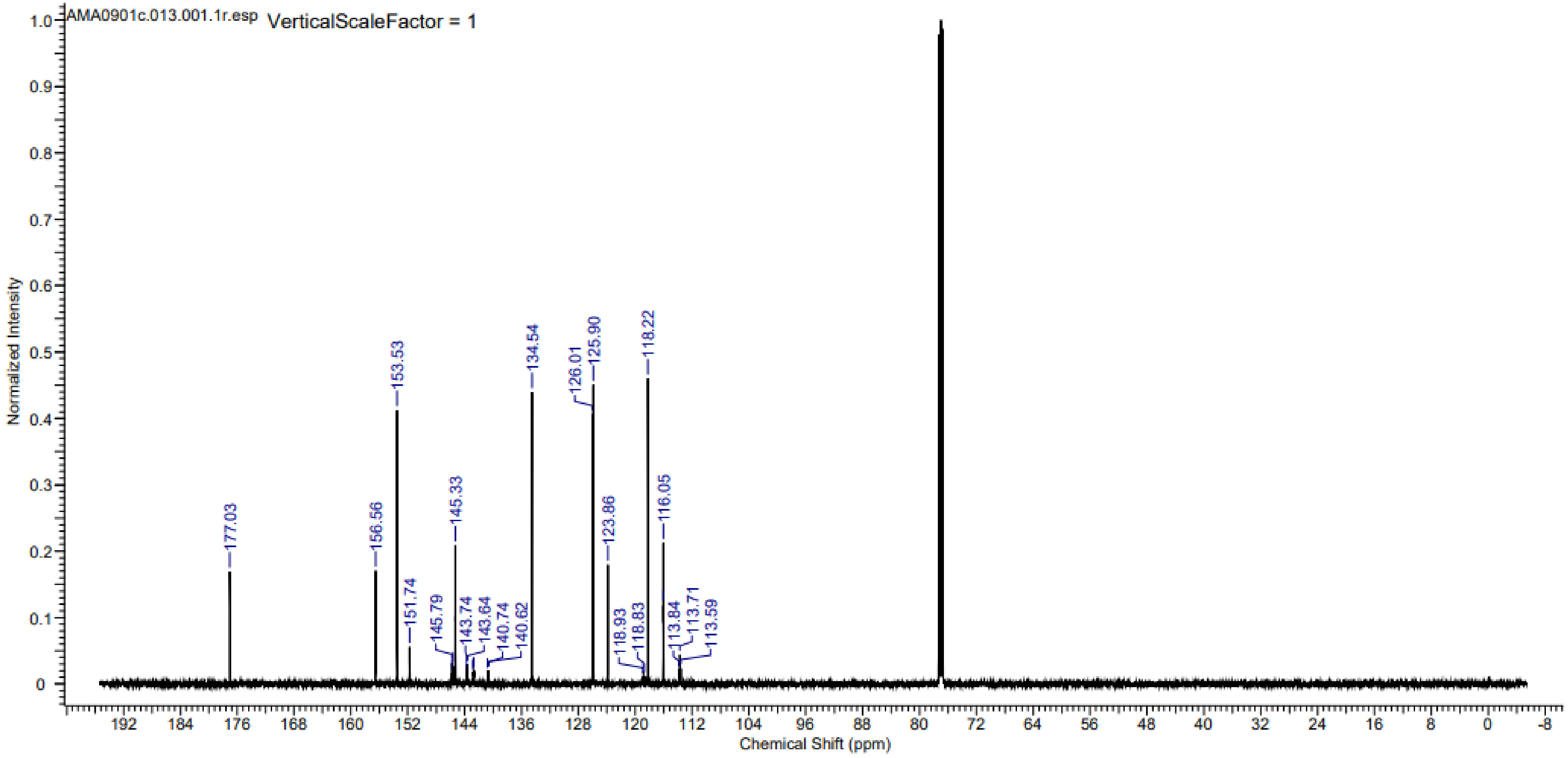

Figure S3. ^{19}F NMR spectrum of compound **4**

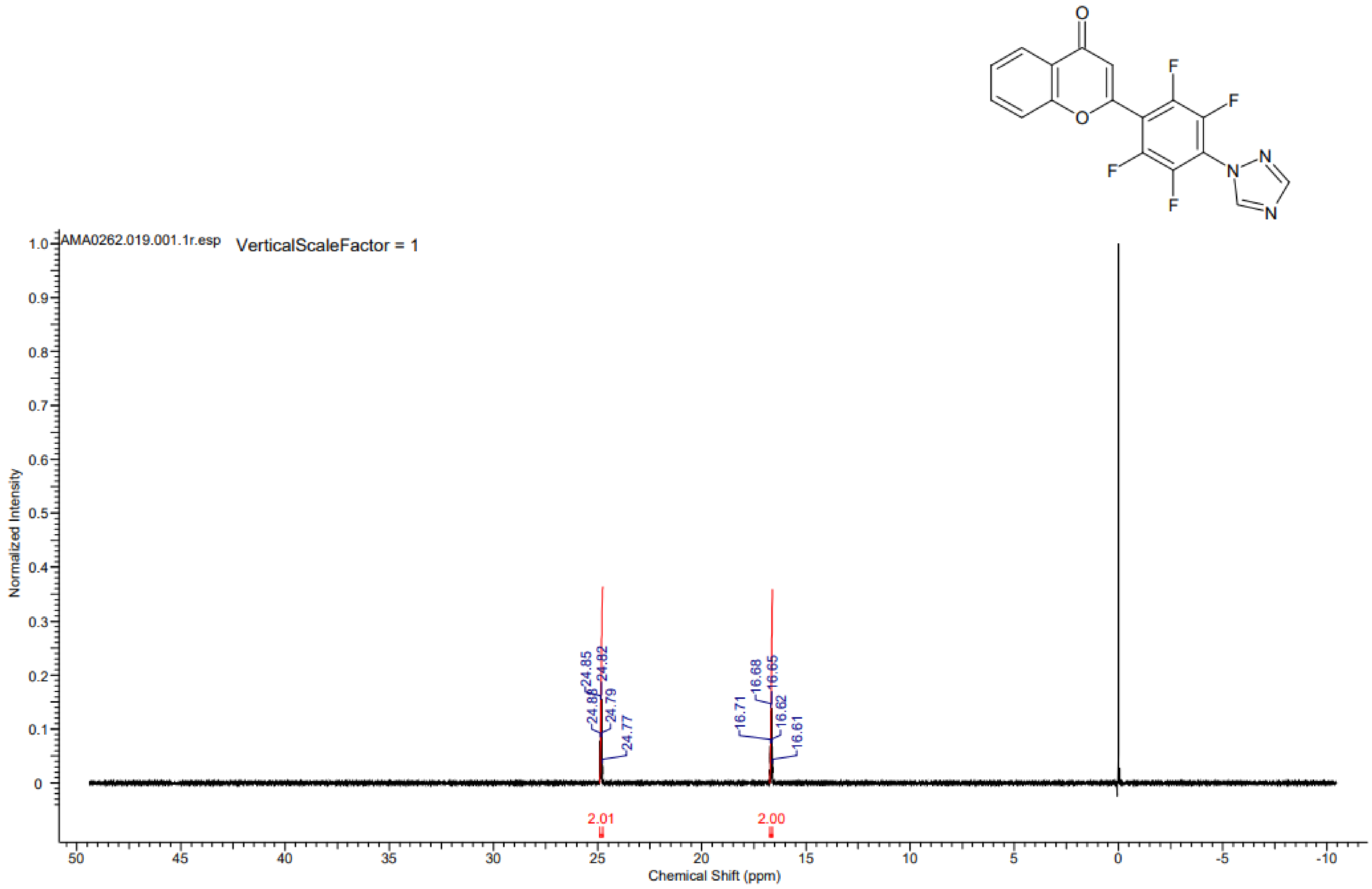

Figure S4. ^{1}H NMR spectrum of compound **7**

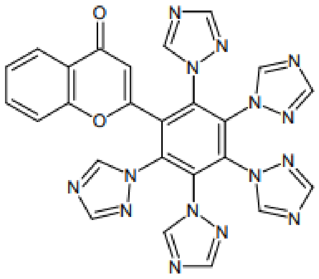

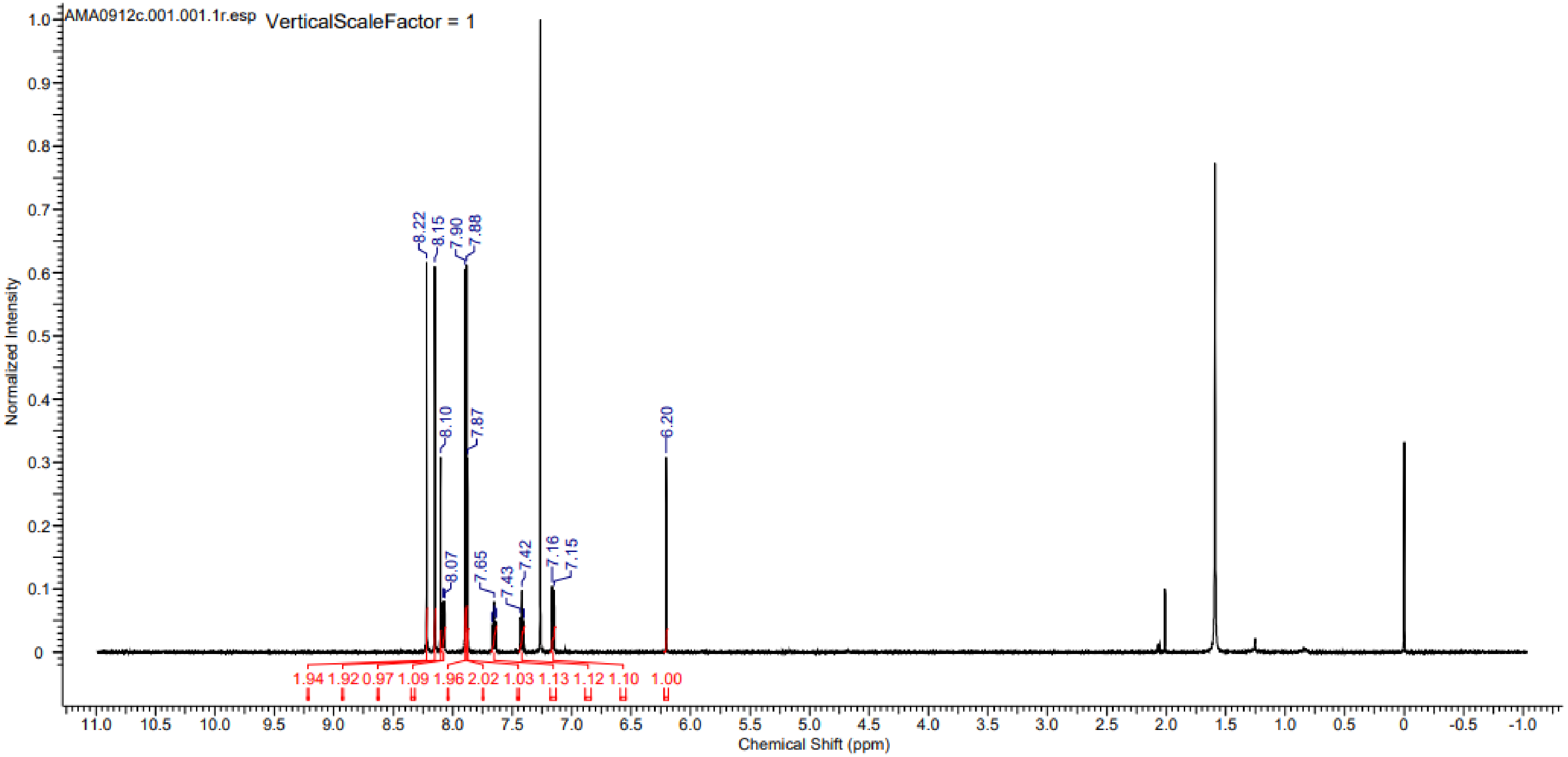

Figure S5. ^{13}C NMR spectrum of compound **7**

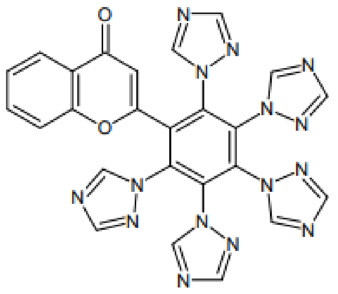

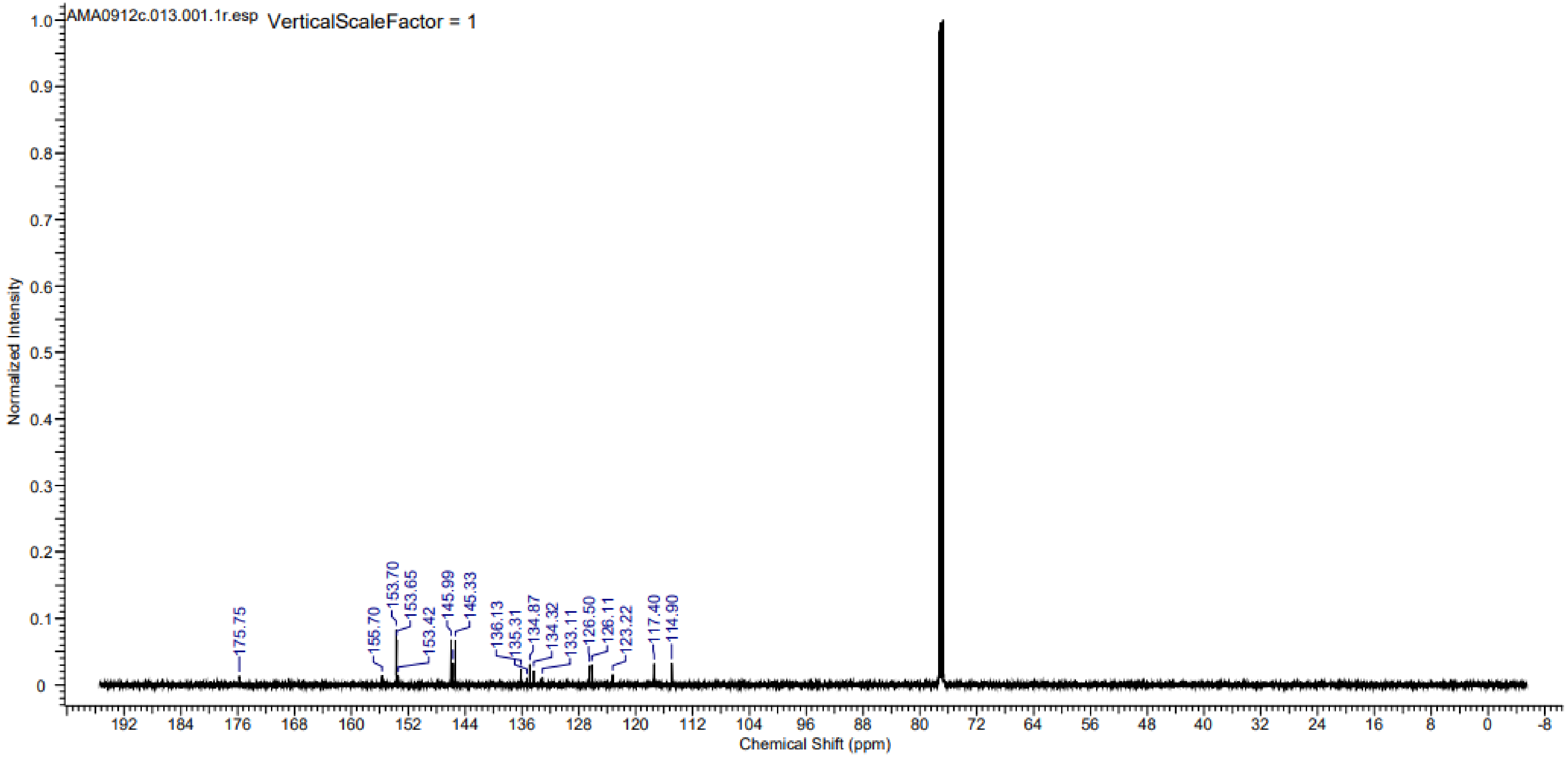

Figure S6. ^{1}H NMR spectrum of compound **8**

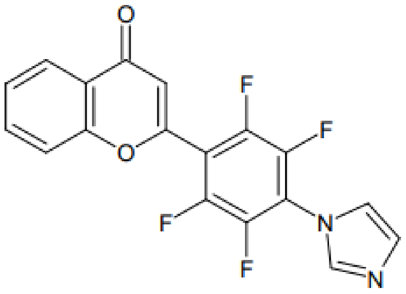

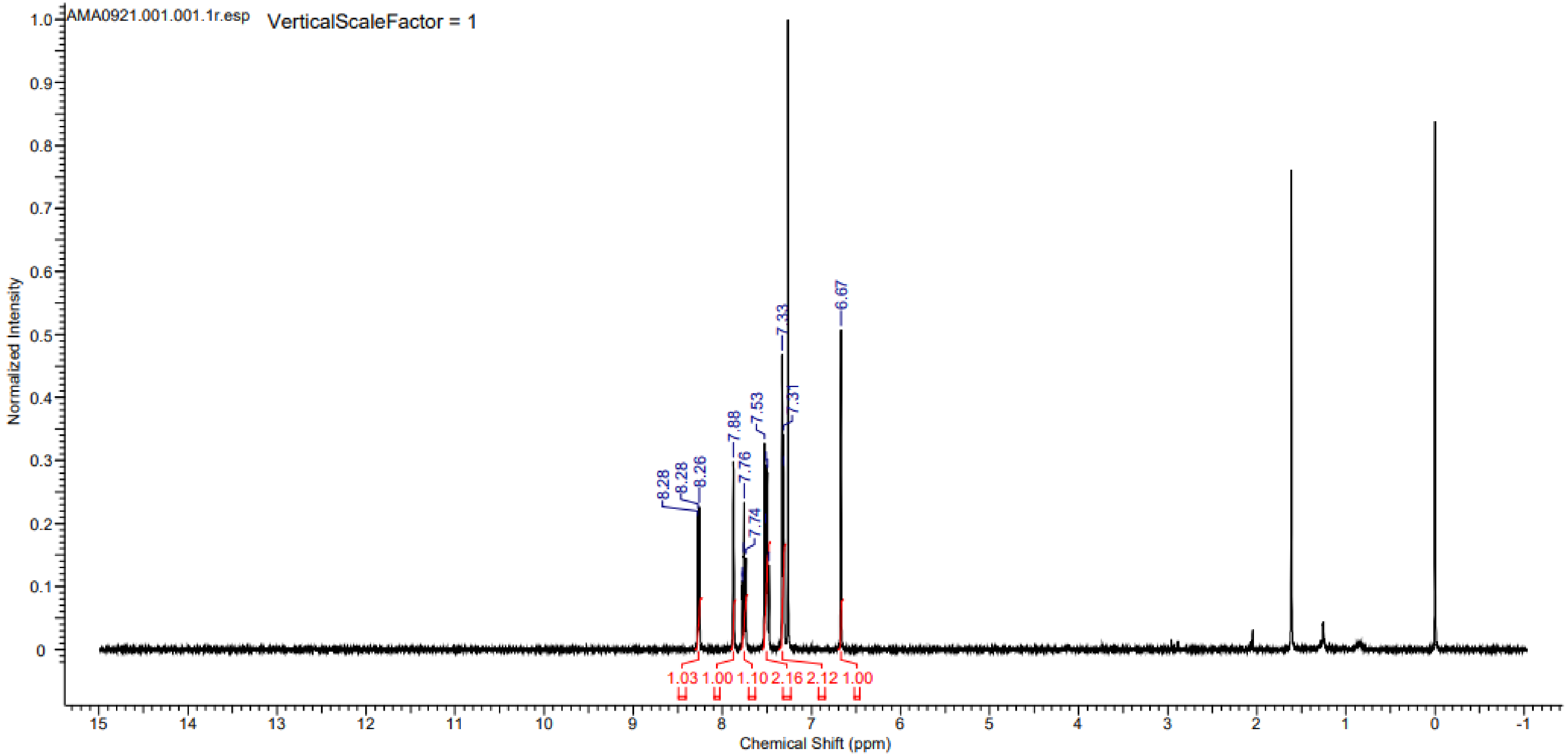

Figure S7. ^{13}C NMR spectrum of compound **8**

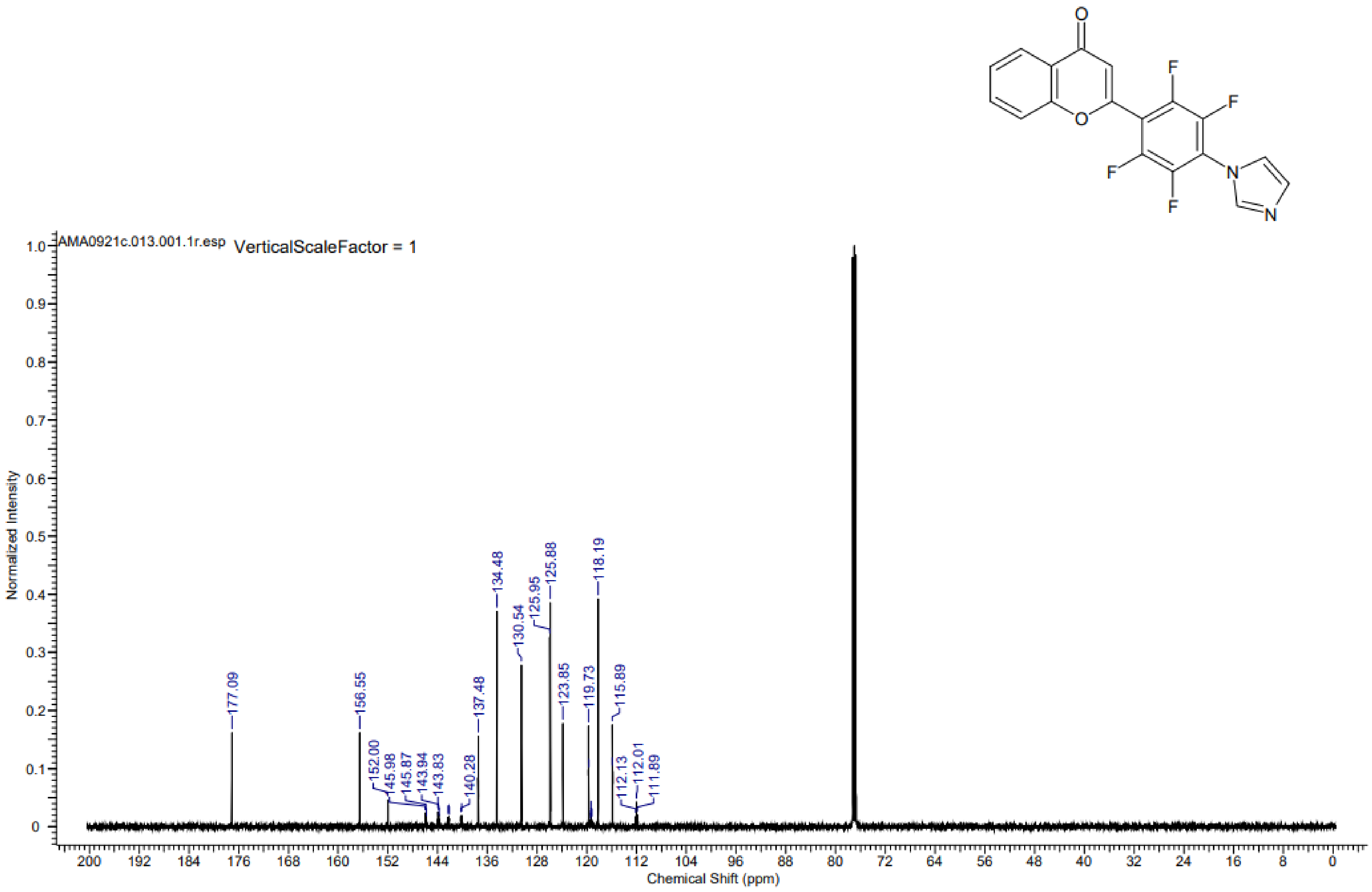

Figure S8. ^{19}F NMR spectrum of compound **8**

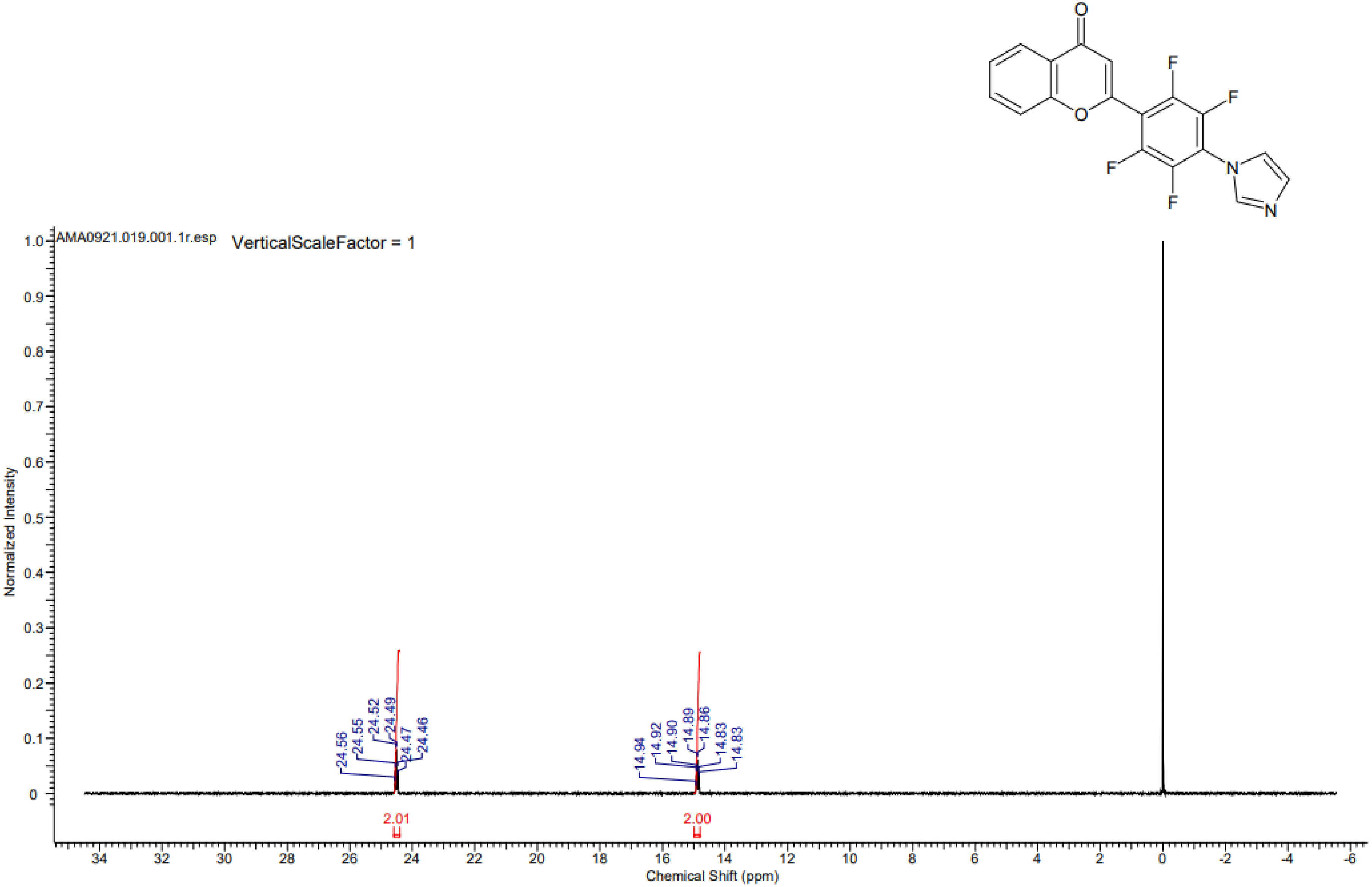

Figure S9. ^{1}H NMR spectrum of compound **9**

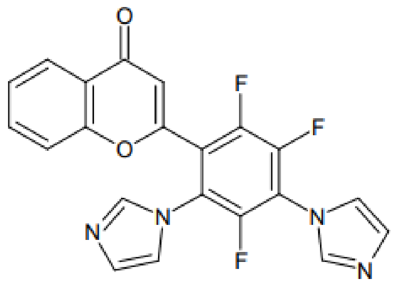

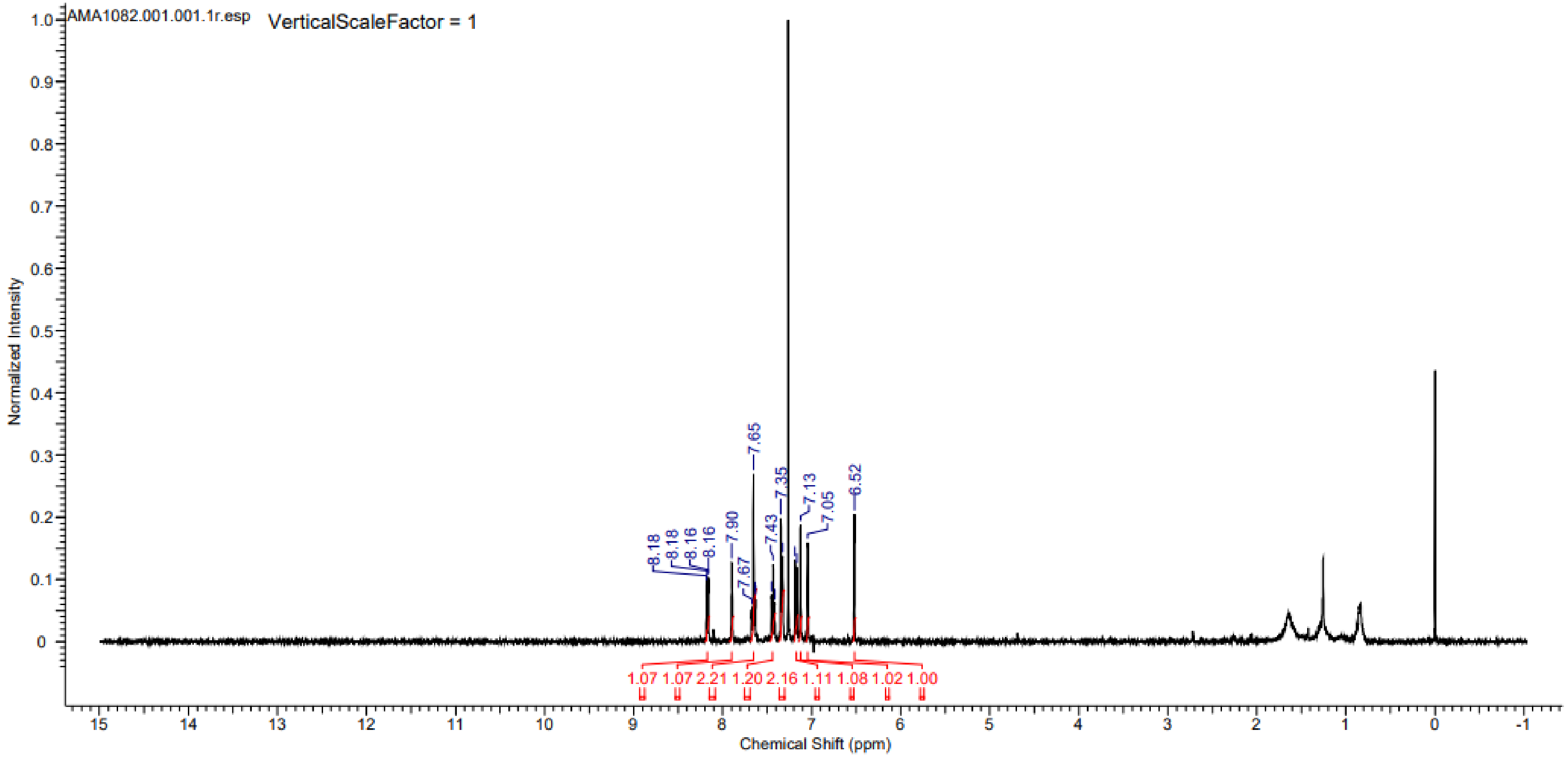

Figure S10. ^{19}F NMR spectrum of compound **9**

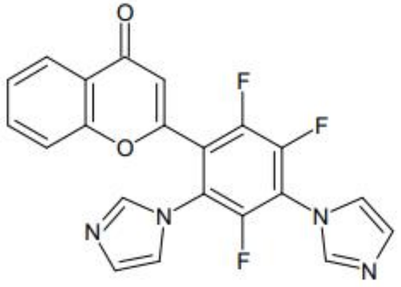

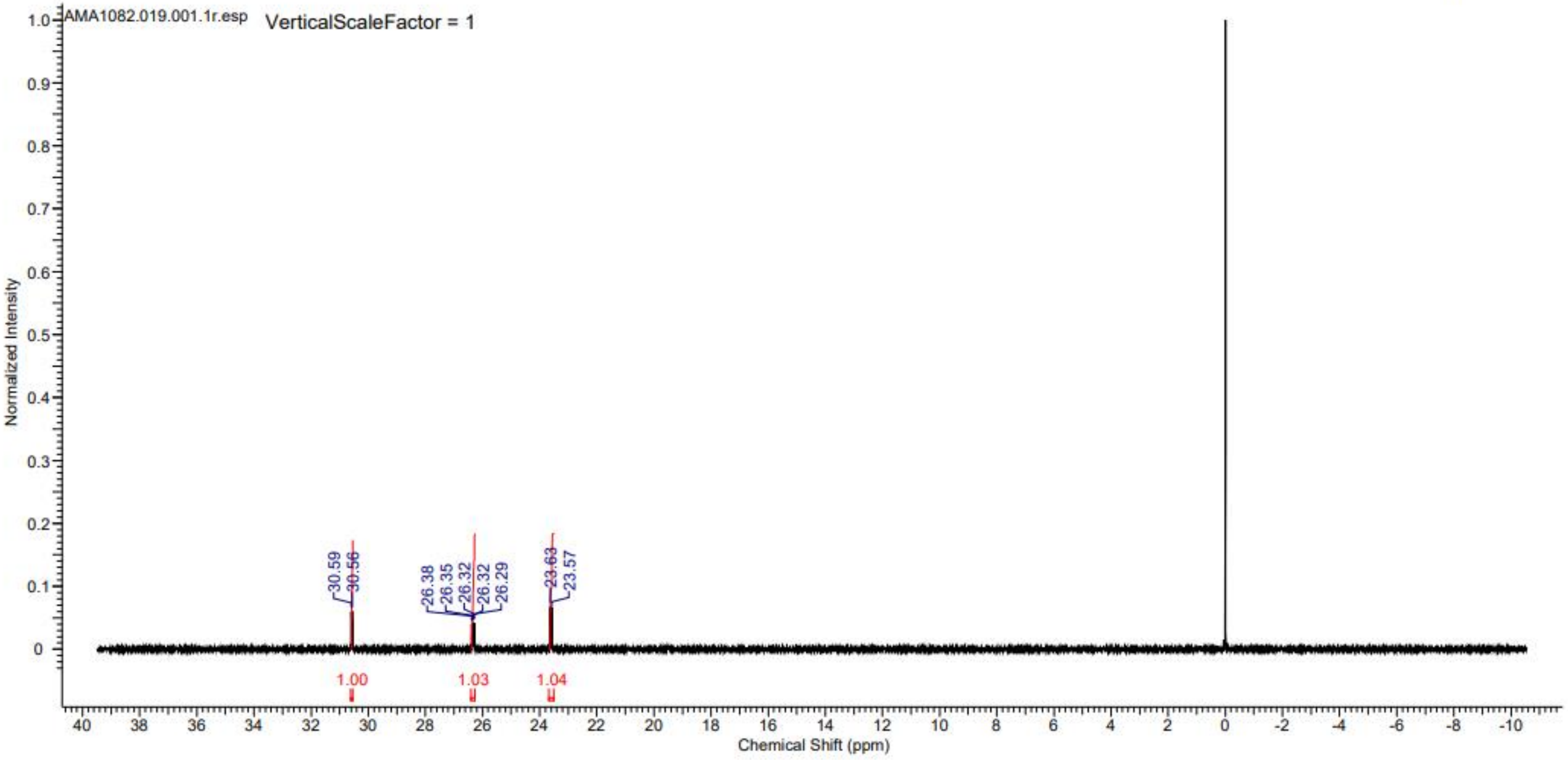

Figure S11. ^{1}H NMR spectrum of compound **10**

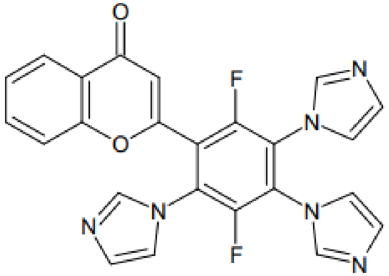

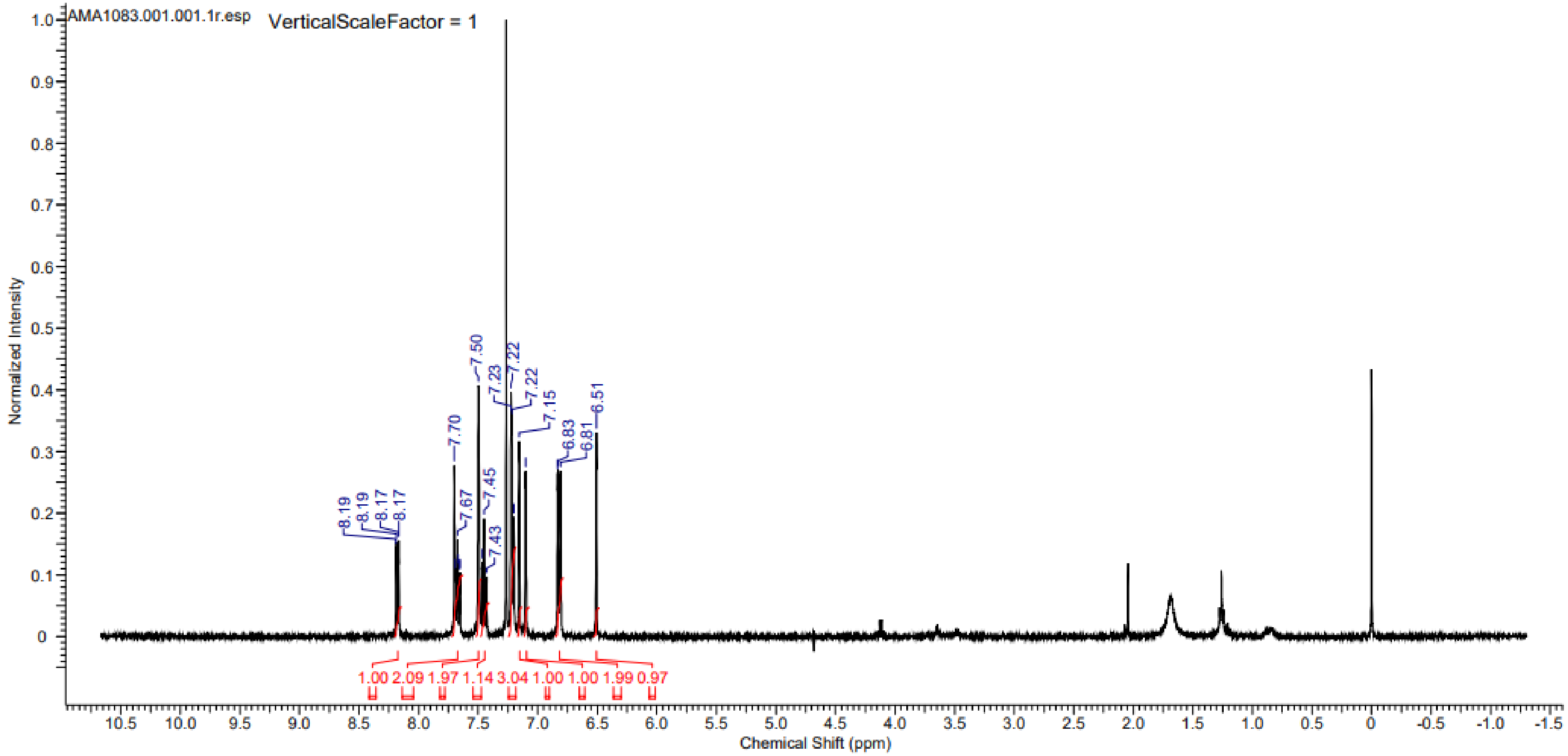

Figure S12. ^{13}C NMR spectrum of compound **10**

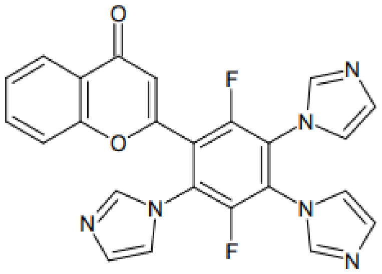

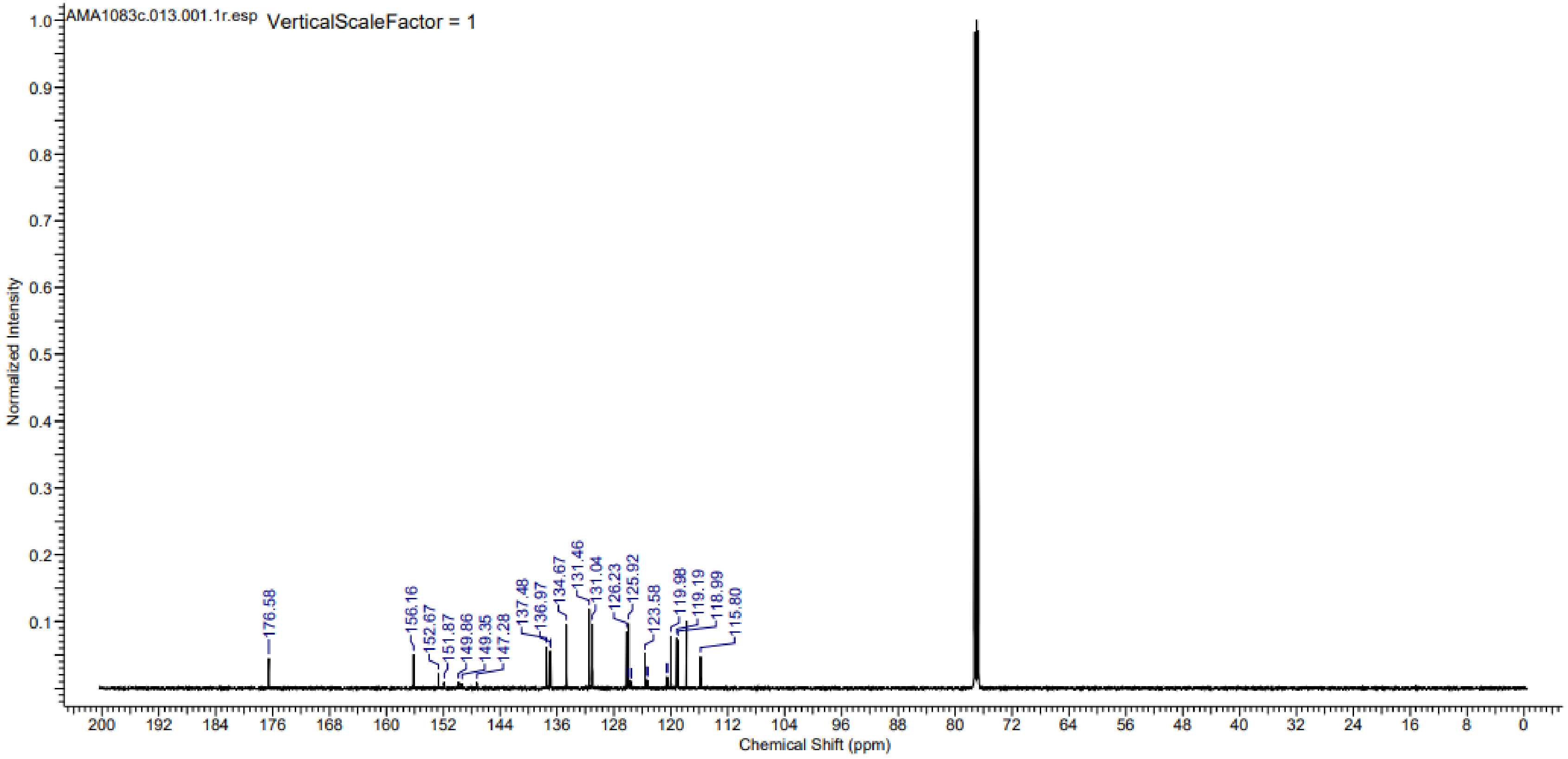

Figure S13. ^{19}F NMR spectrum of compound **10**

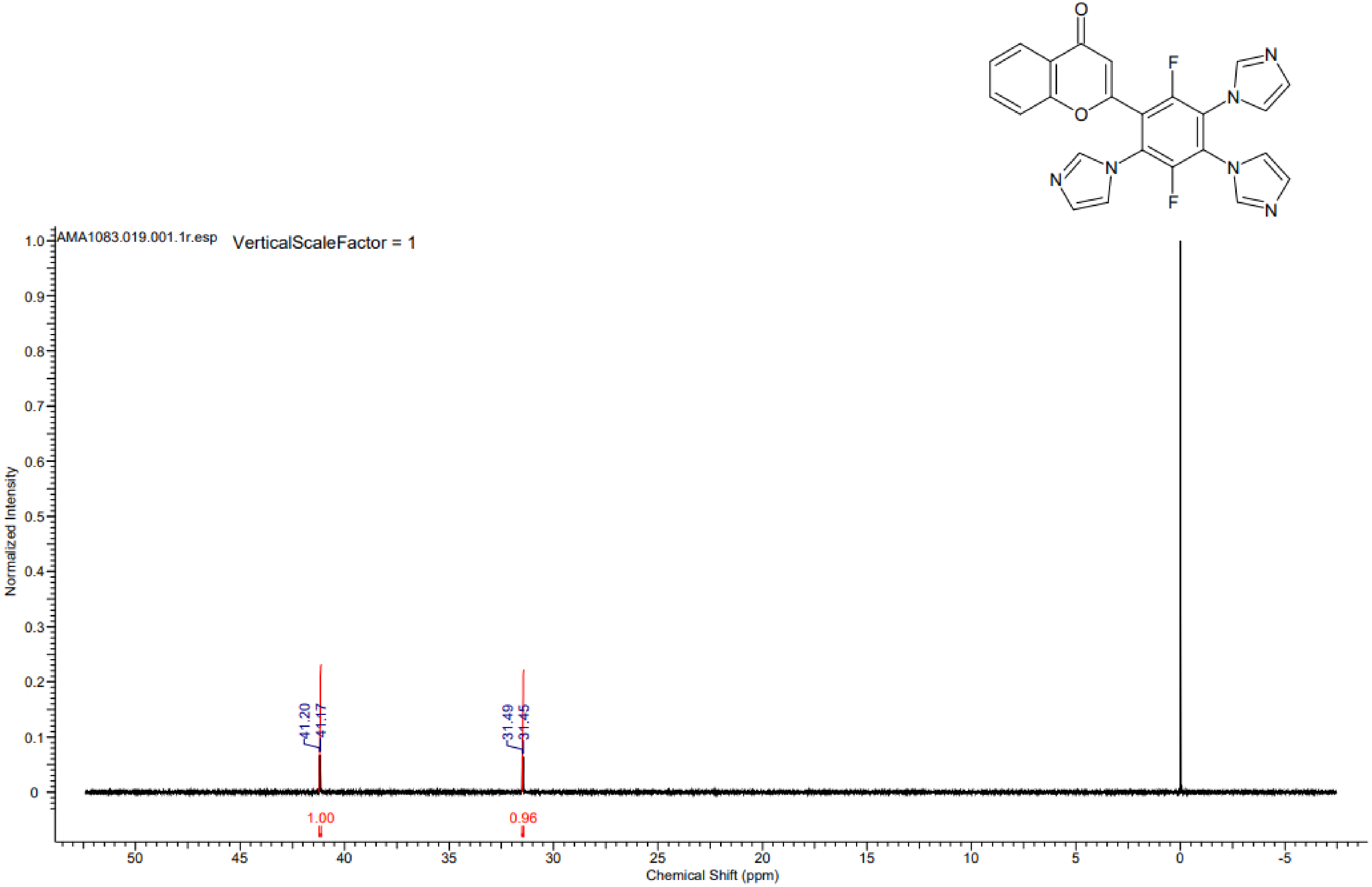

Figure S14. ^{1}H NMR spectrum of compound **11**

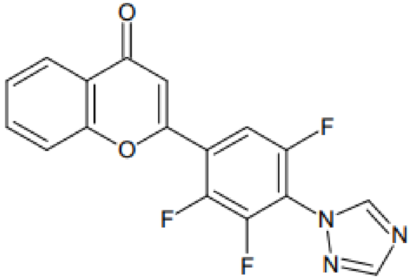

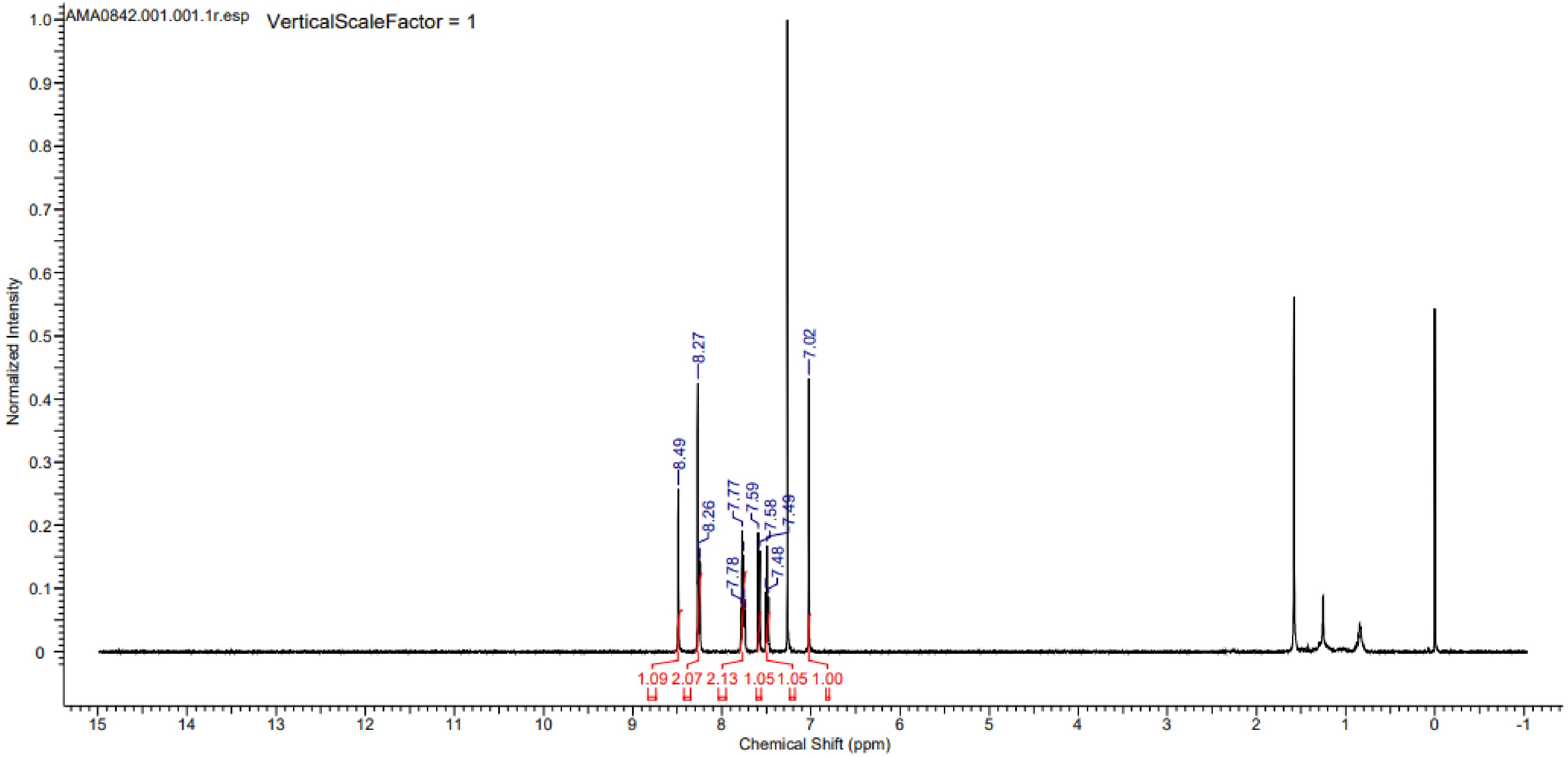

Figure S15. ^{13}C NMR spectrum of compound **11**

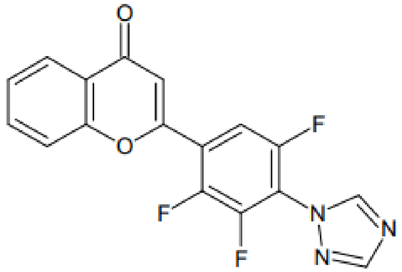

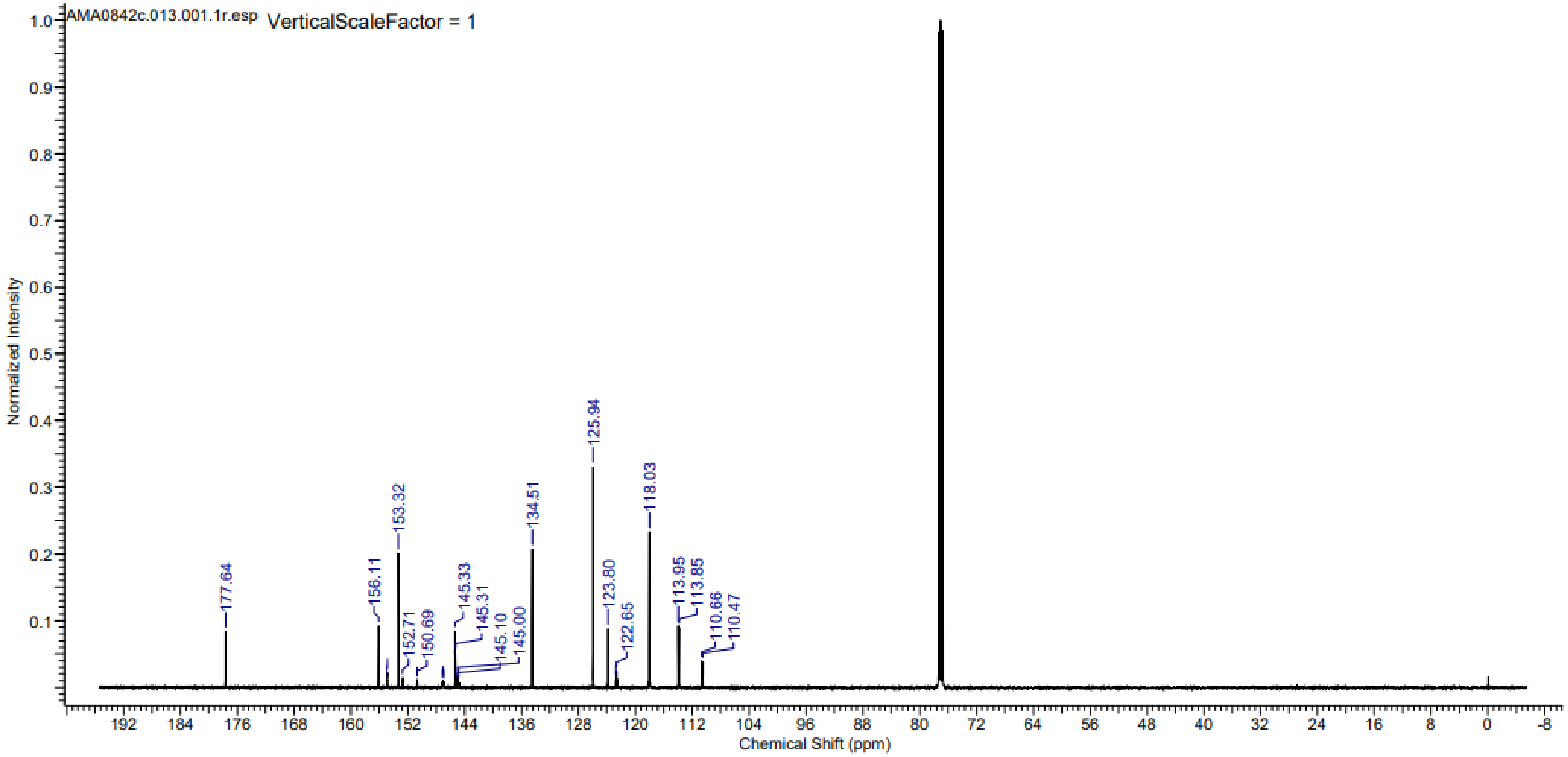

Figure S16. ^{19}F NMR spectrum of compound **11**

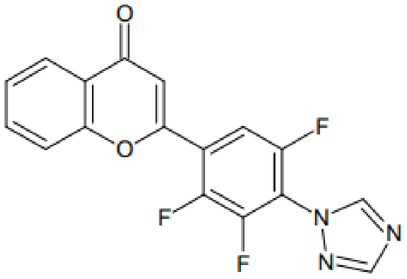

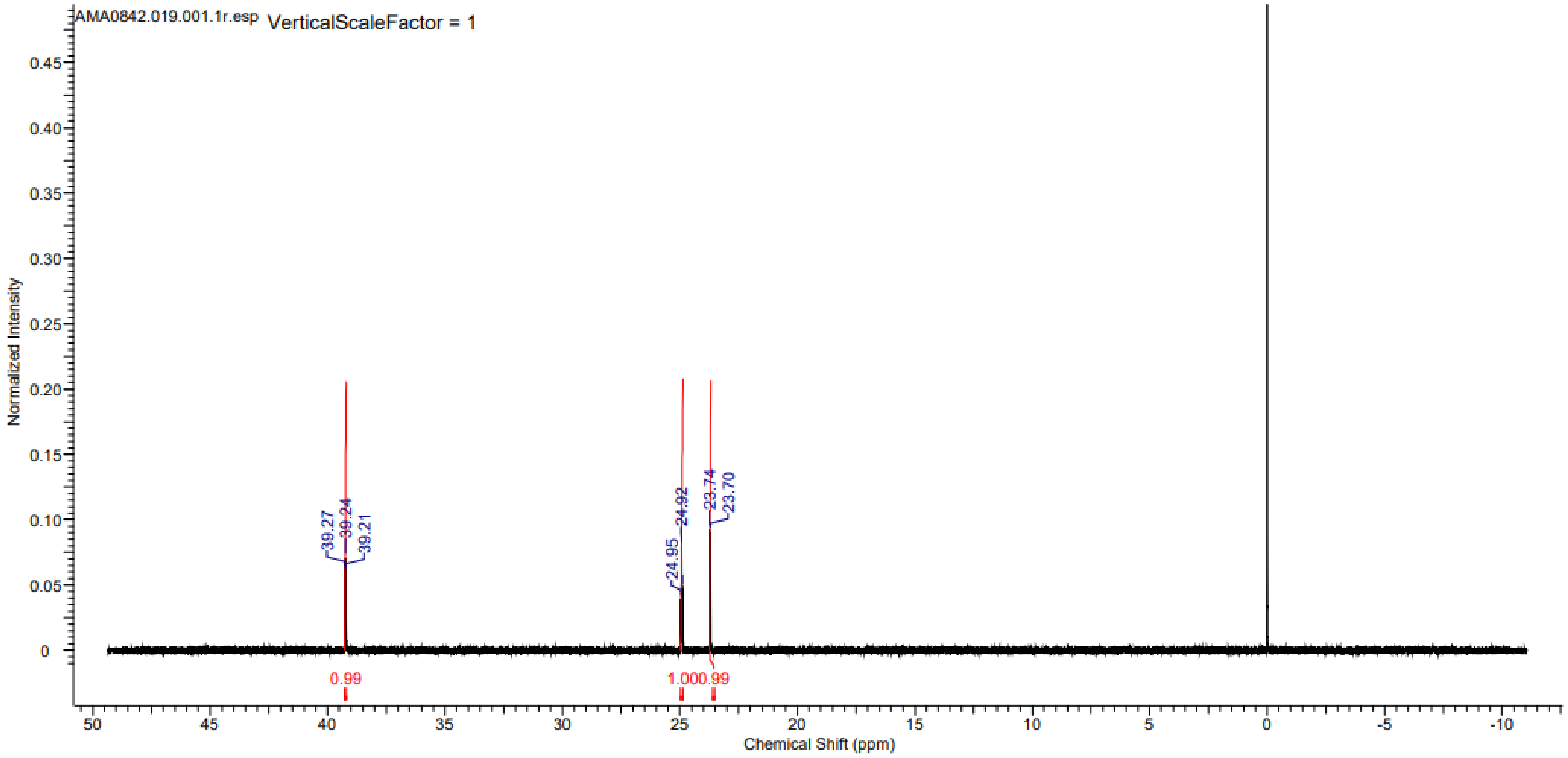

Figure S17. ^{1}H NMR spectrum of compound **14**

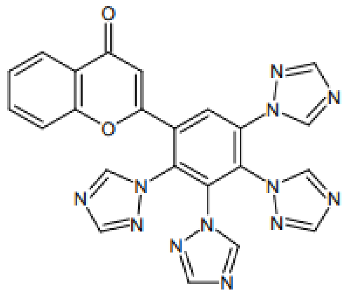

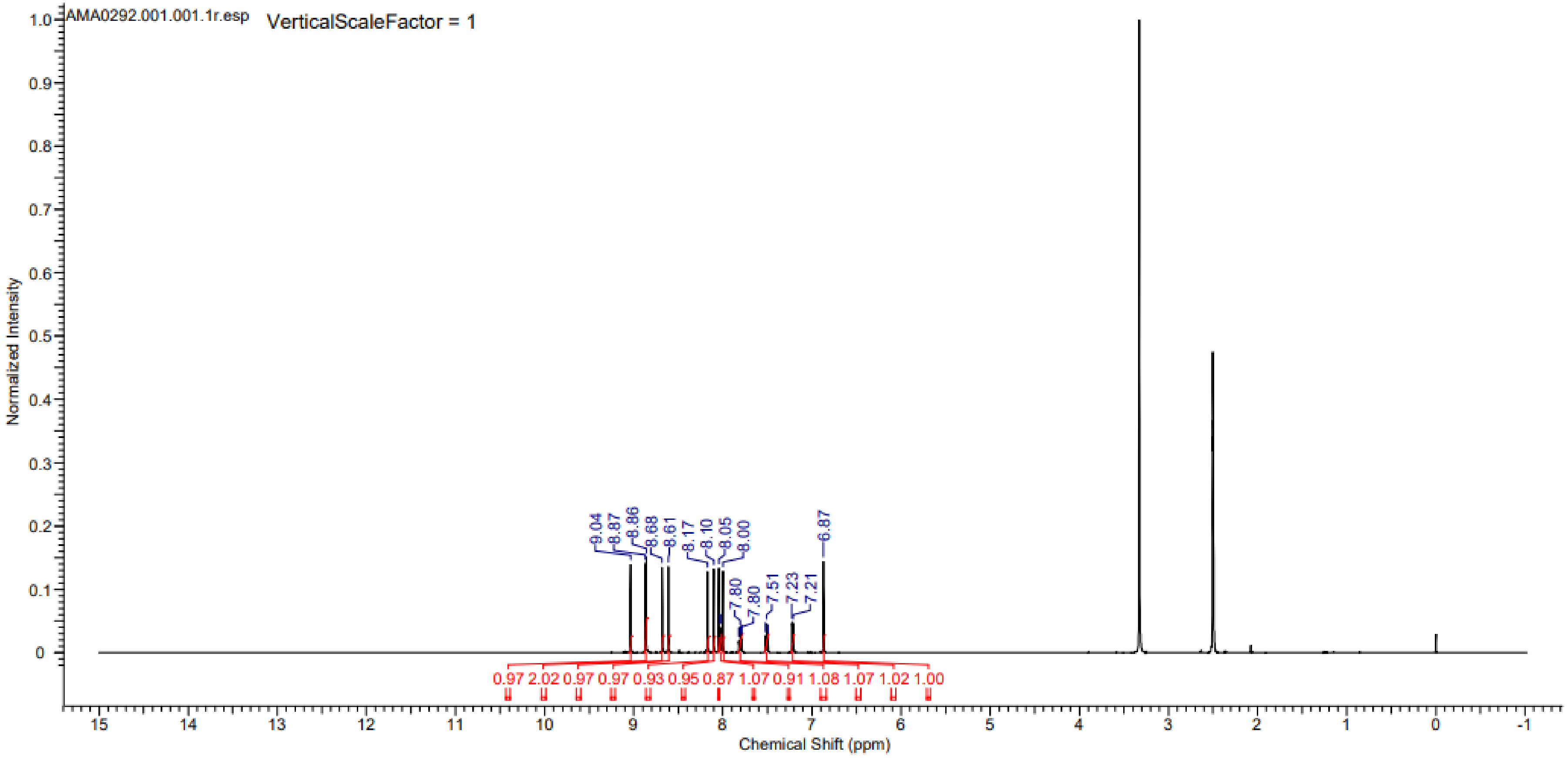

Figure S18. ^{13}C NMR spectrum of compound **14**

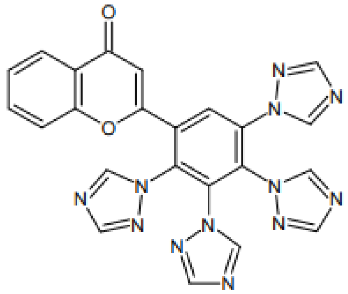

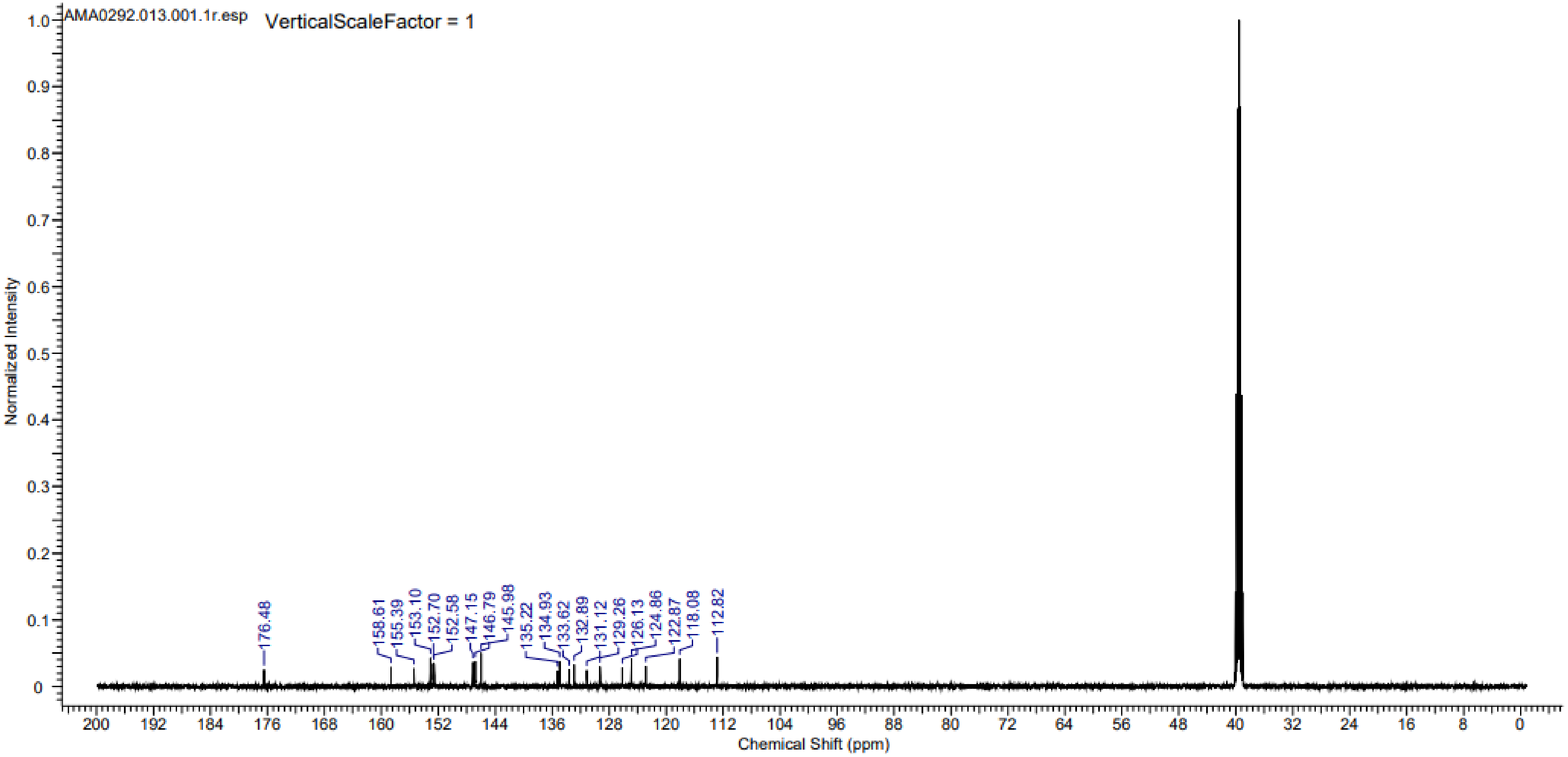

Figure S19. ^{1}H NMR spectrum of compound **15**

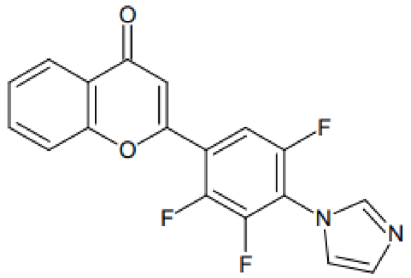

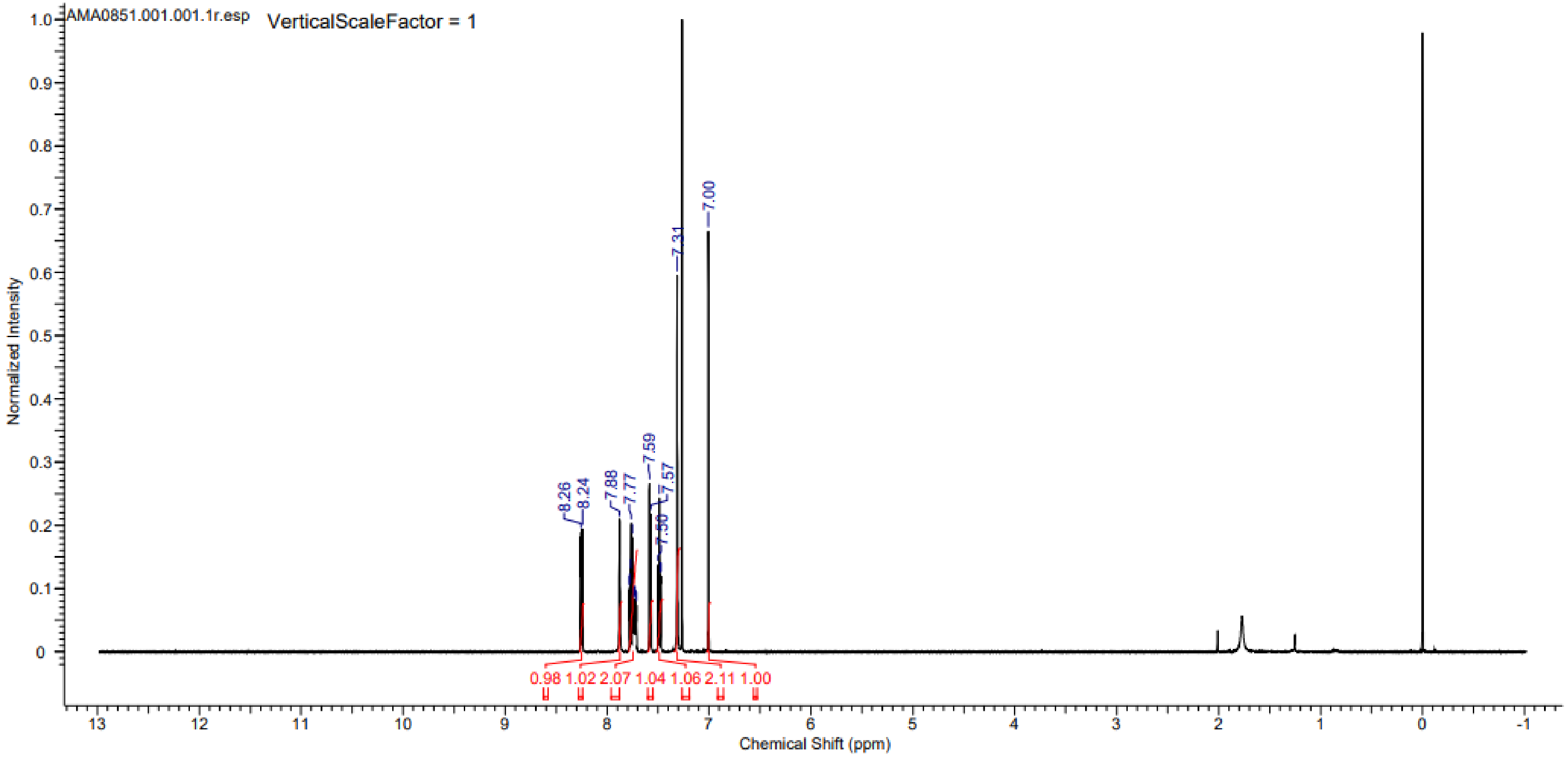

Figure S20. ^{13}C NMR spectrum of compound **15**

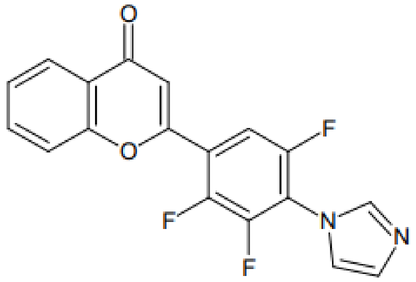

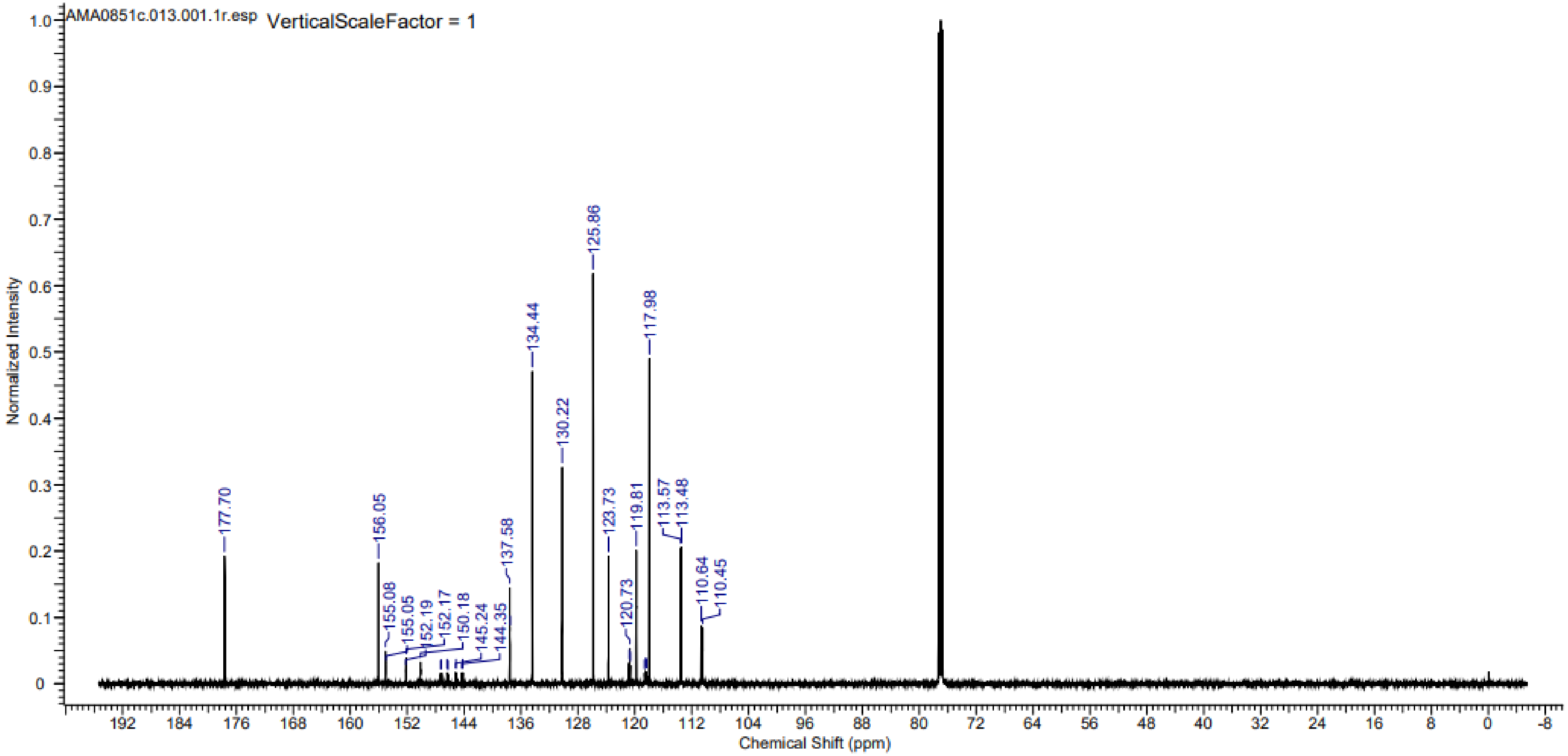

Figure S21. ^{19}F NMR spectrum of compound **15**

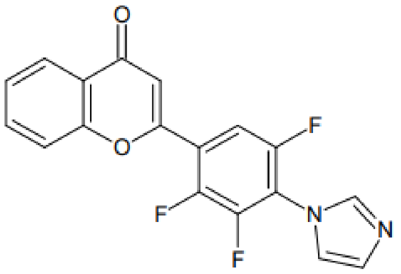

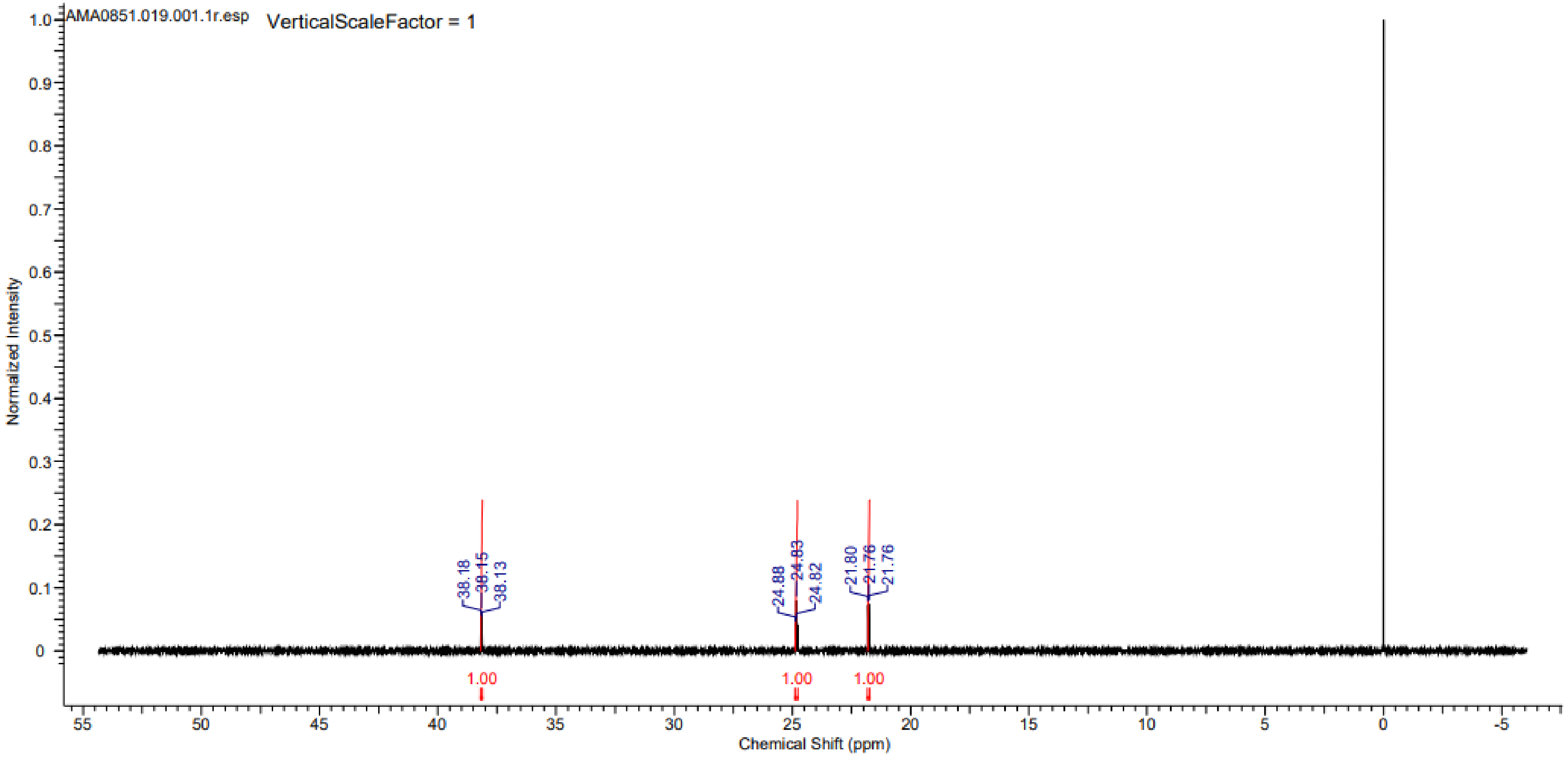

Figure S22. ^{1}H NMR spectrum of compound **17**

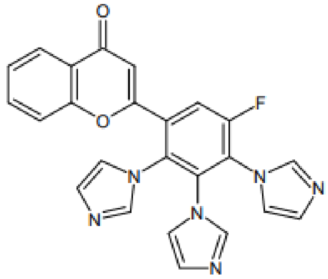

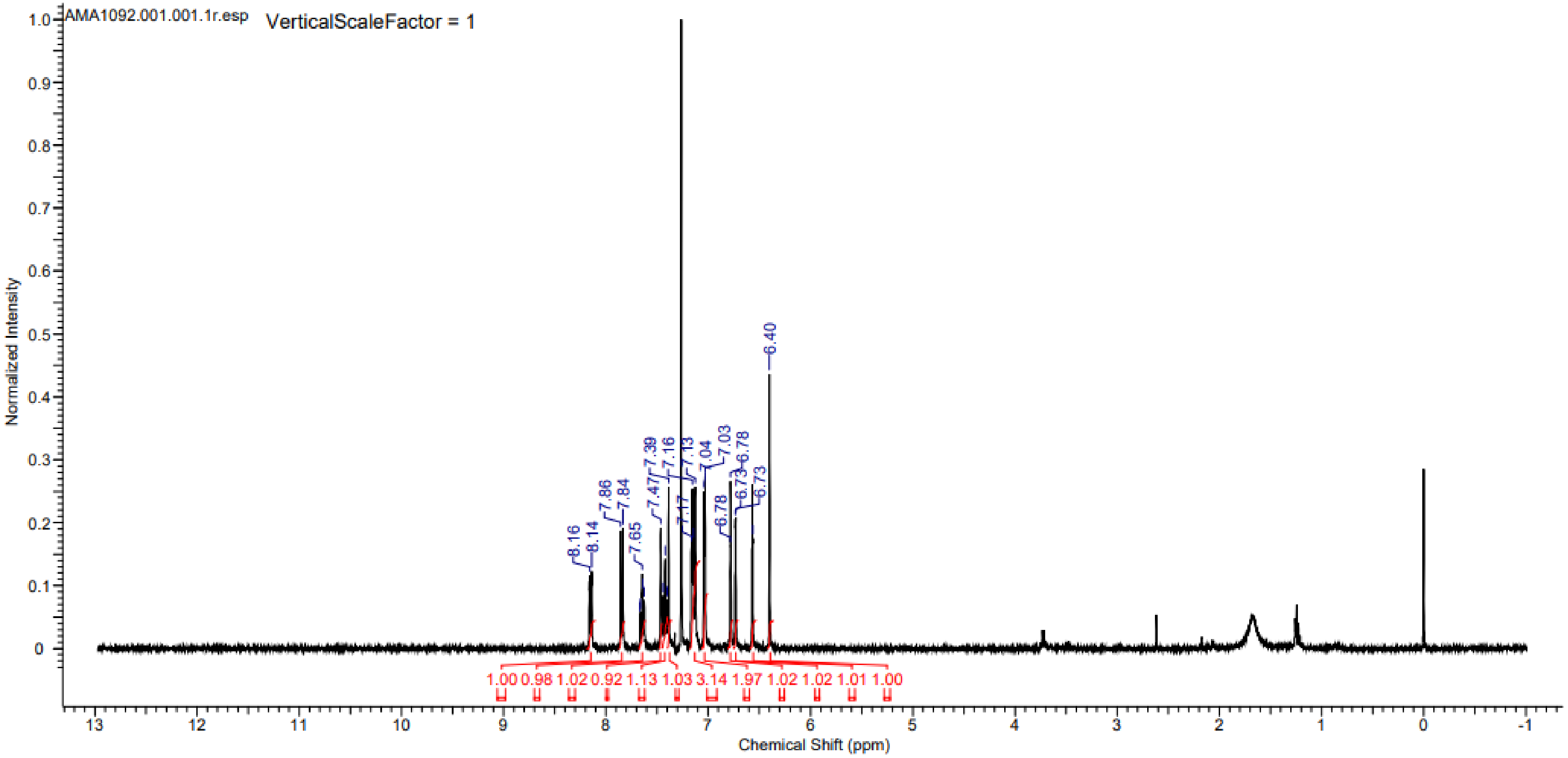

Figure S23. ^{13}C NMR spectrum of compound **17**

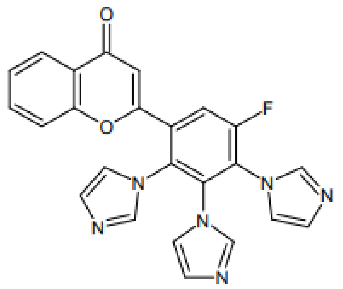

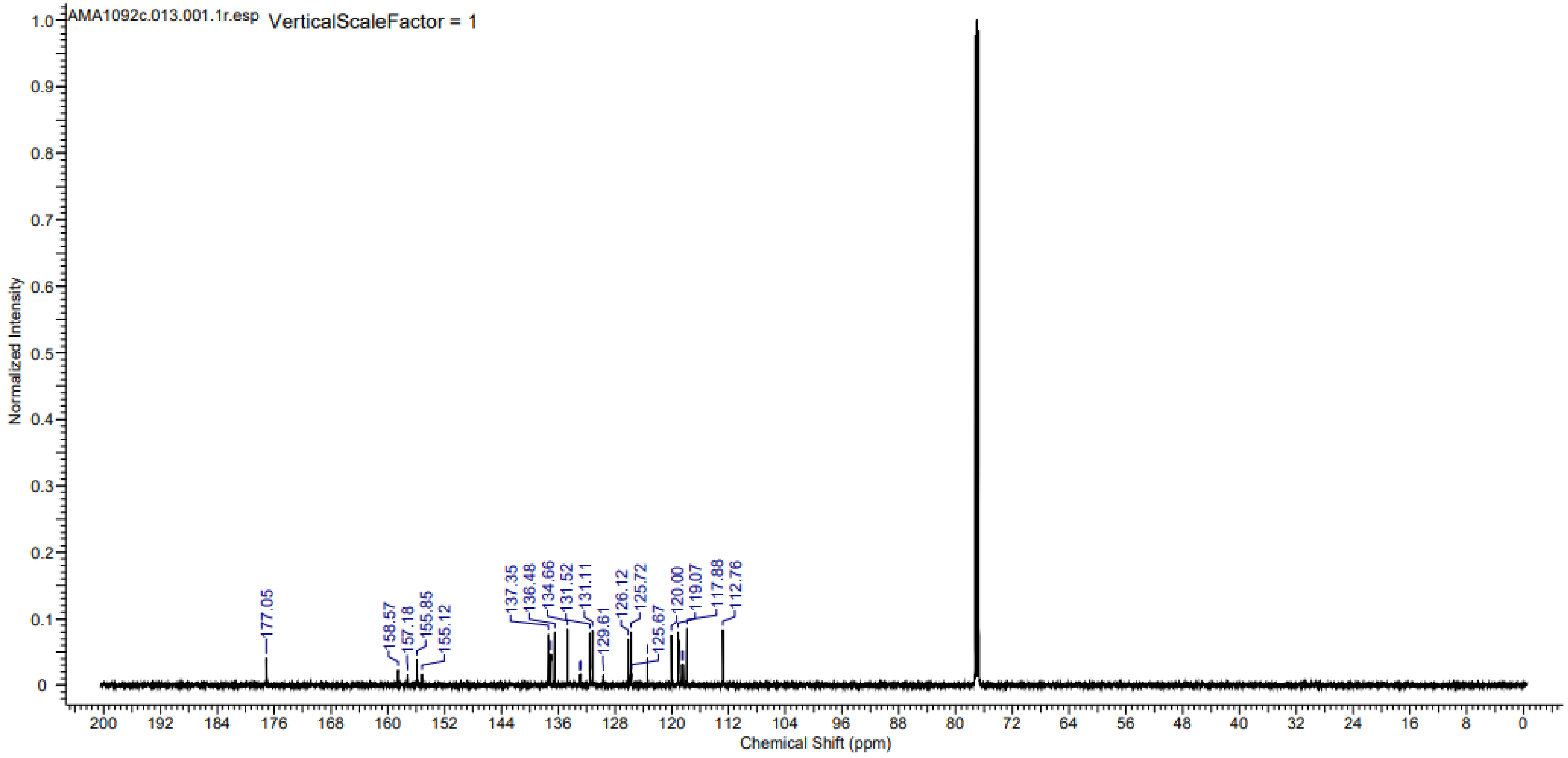

Figure S24. ^{19}F NMR spectrum of compound **17**

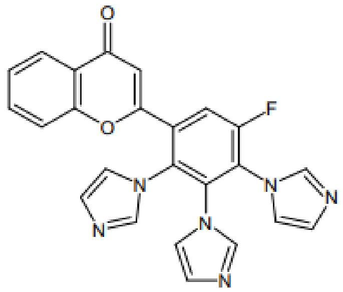

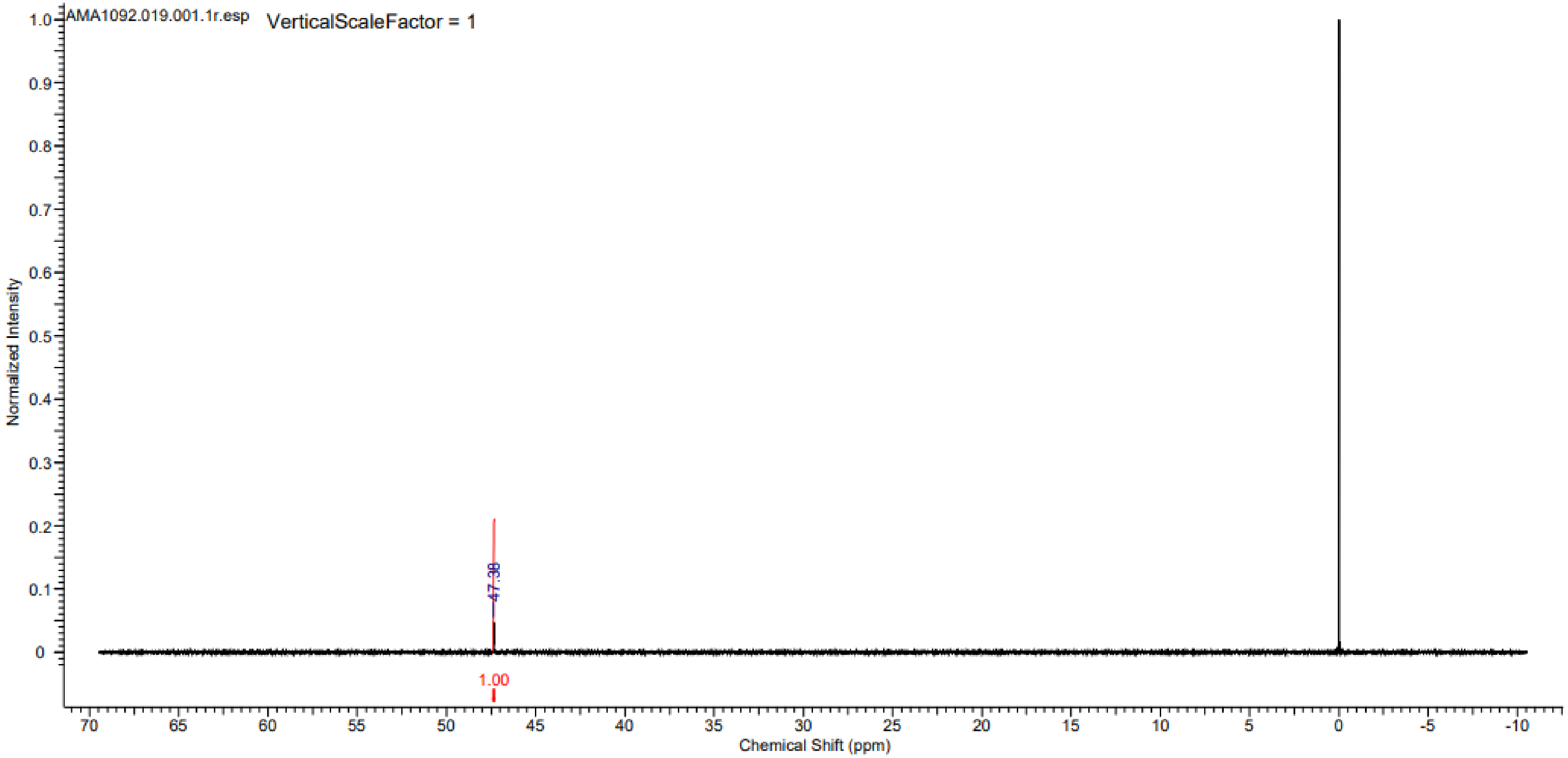

Figure S25. ^{1}H NMR spectrum of compound **18**

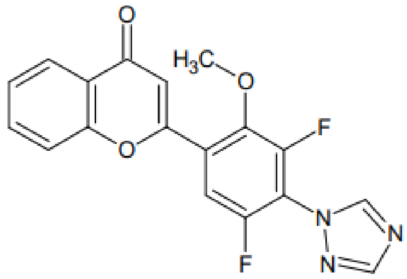

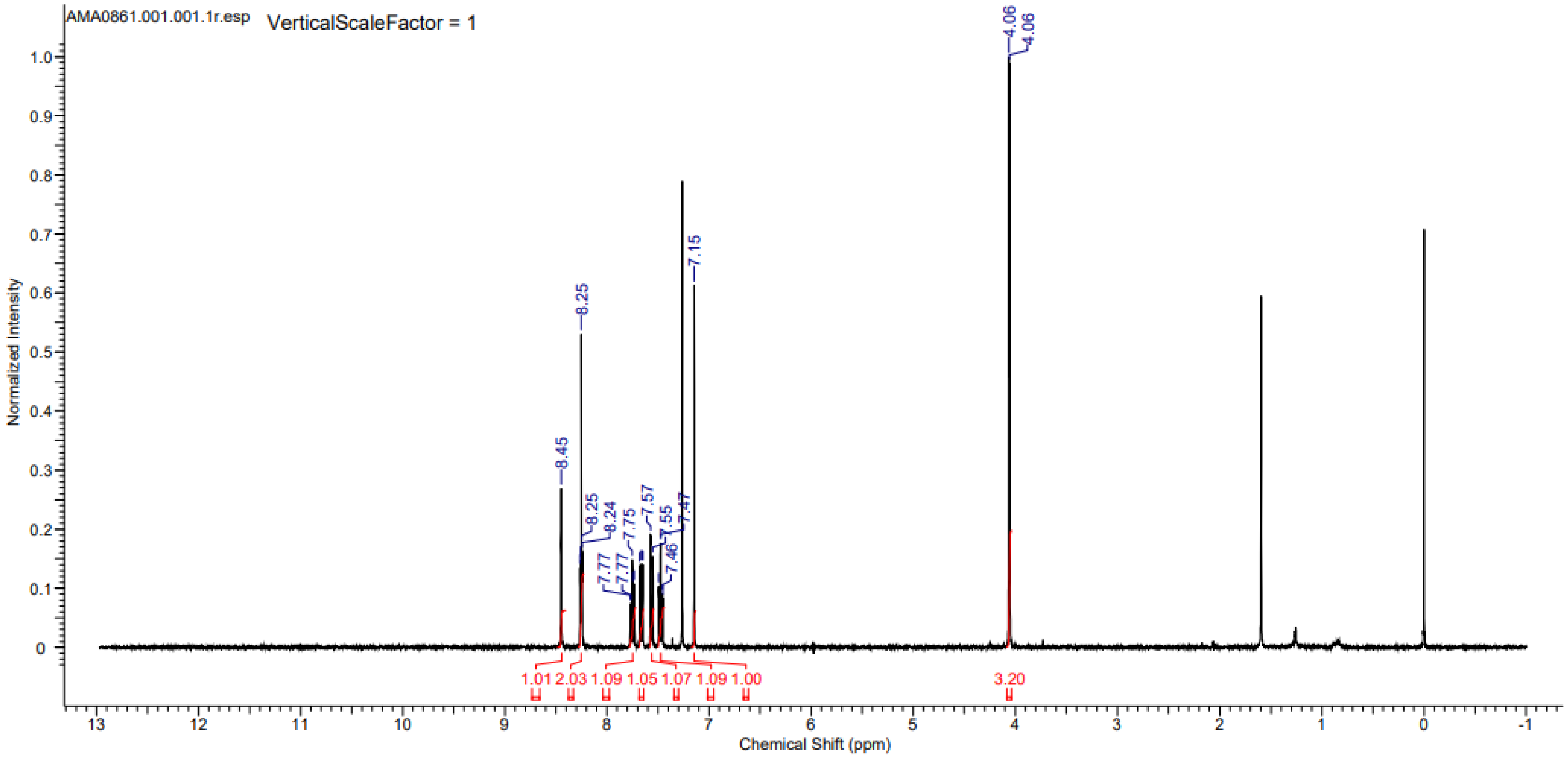

Figure S26. ^{13}C NMR spectrum of compound **18**

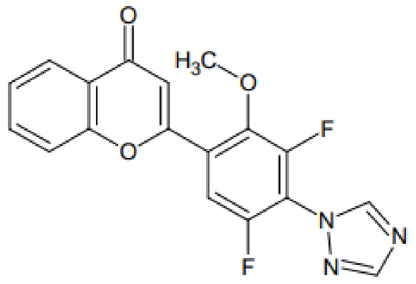

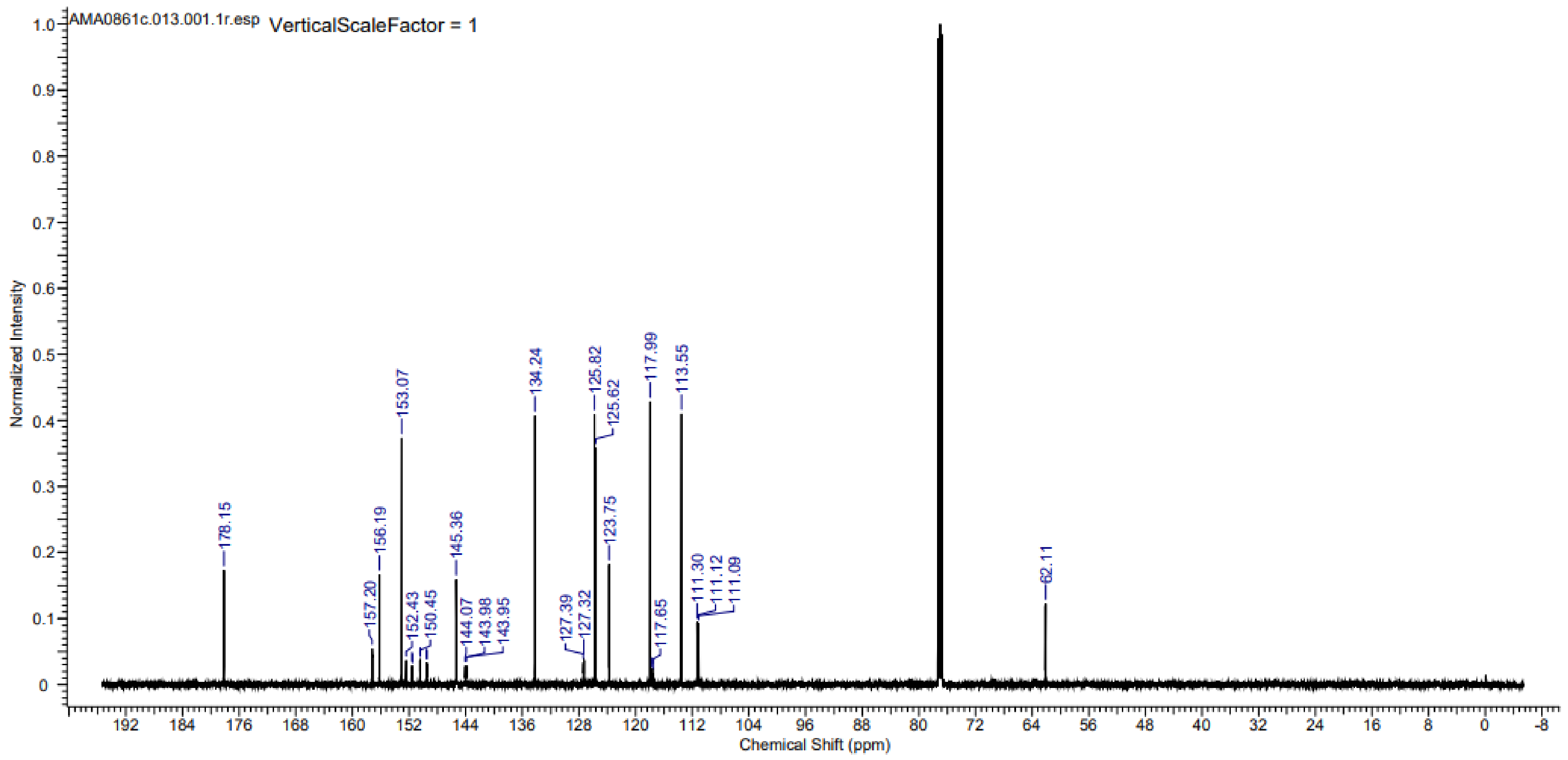

Figure S27. ^{19}F NMR spectrum of compound **18**

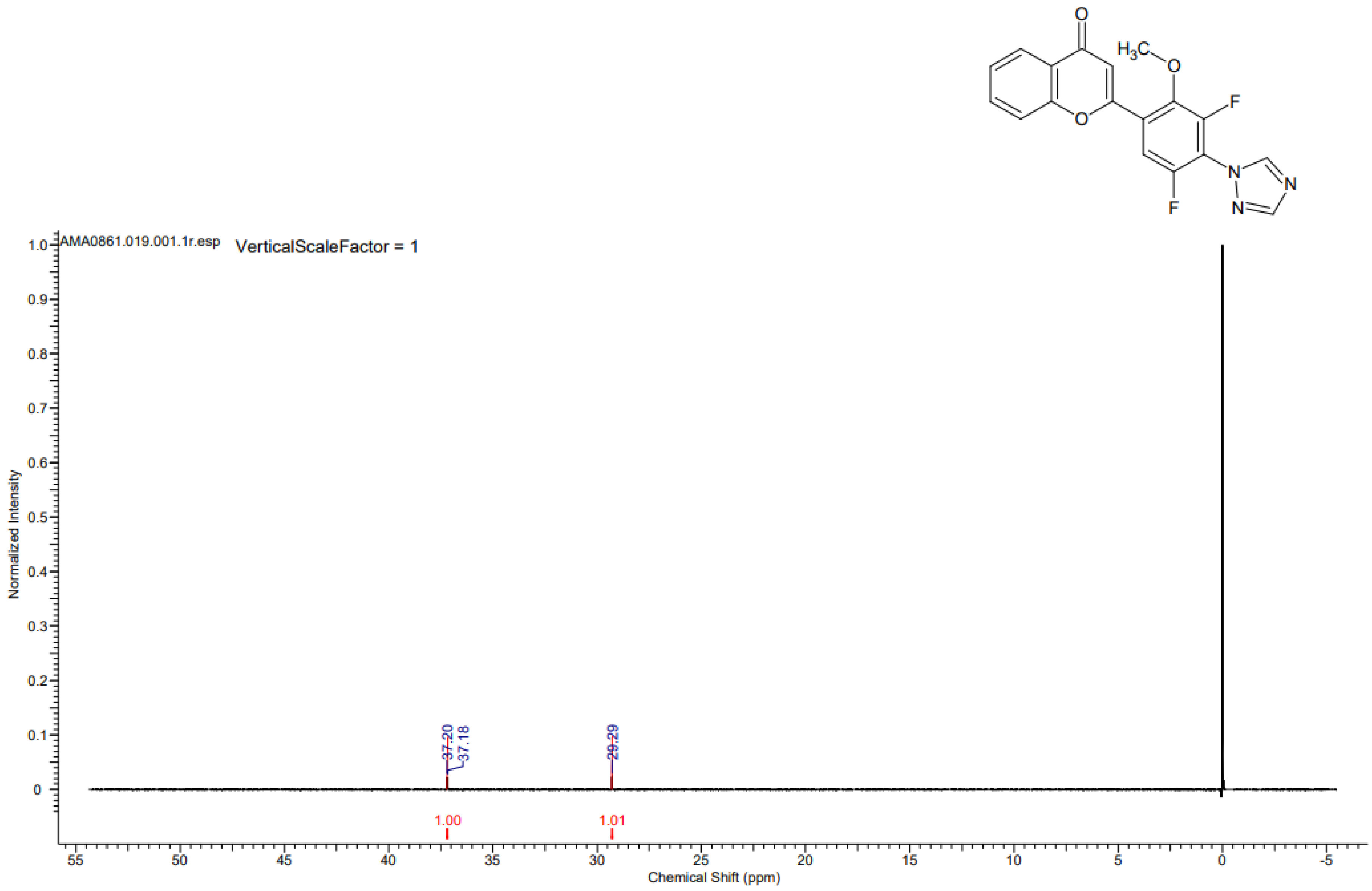

Figure S28. ^{1}H NMR spectrum of compound **19**

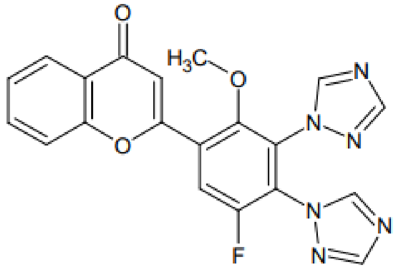

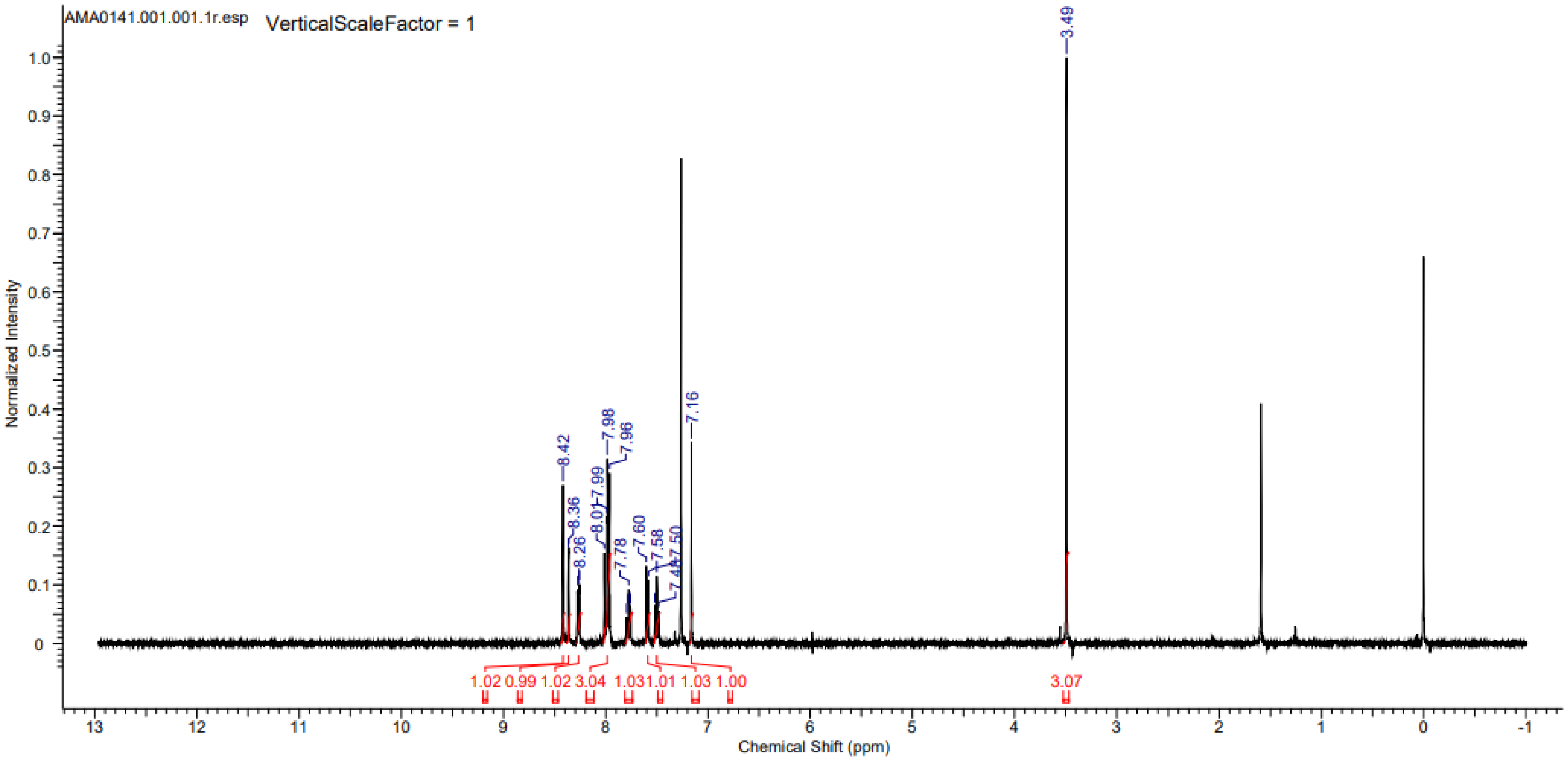

Figure S29. ^{13}C NMR spectrum of compound **19**

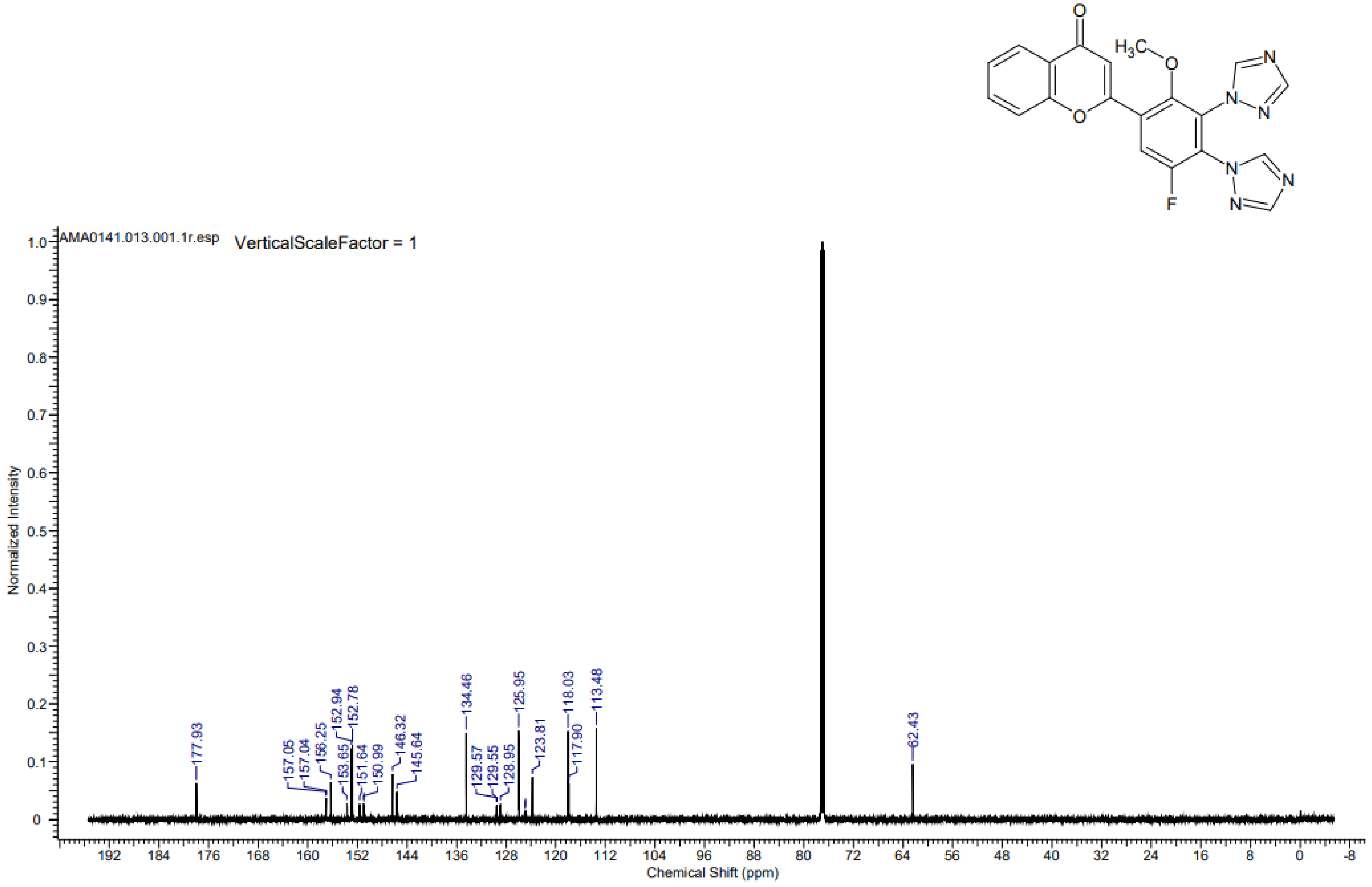

Figure S30. ^{19}F NMR spectrum of compound **19**

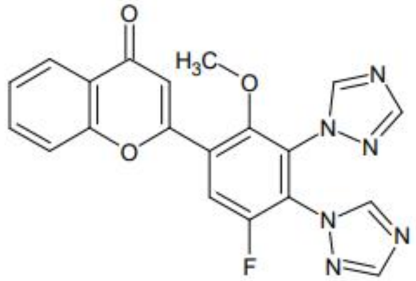

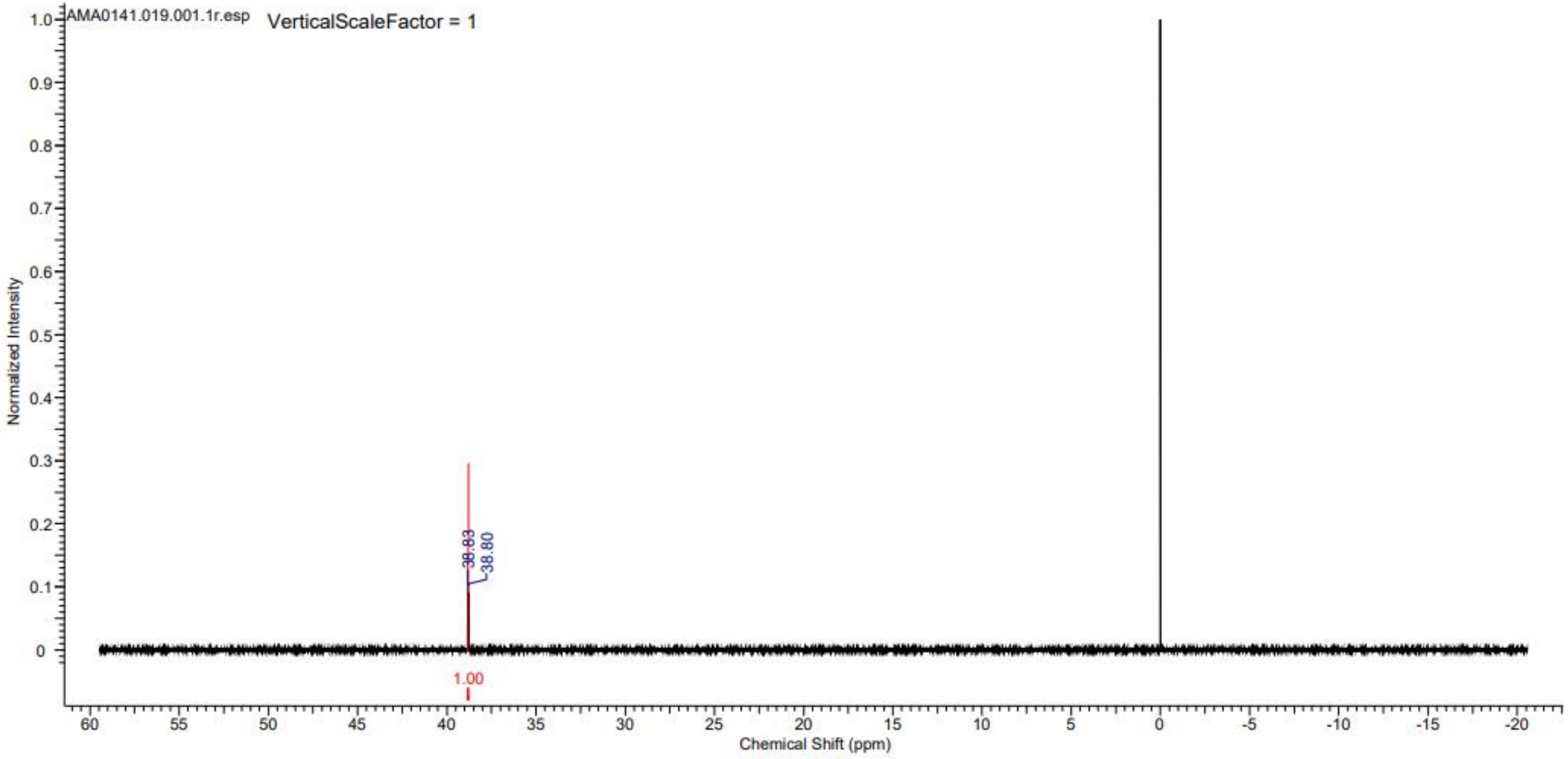

Figure S31. ^{1}H NMR spectrum of compound **22**

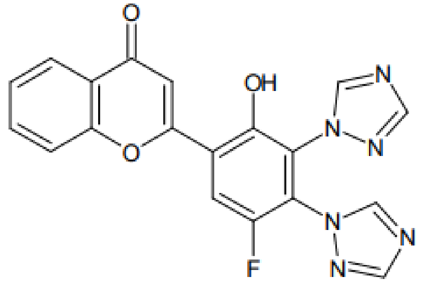

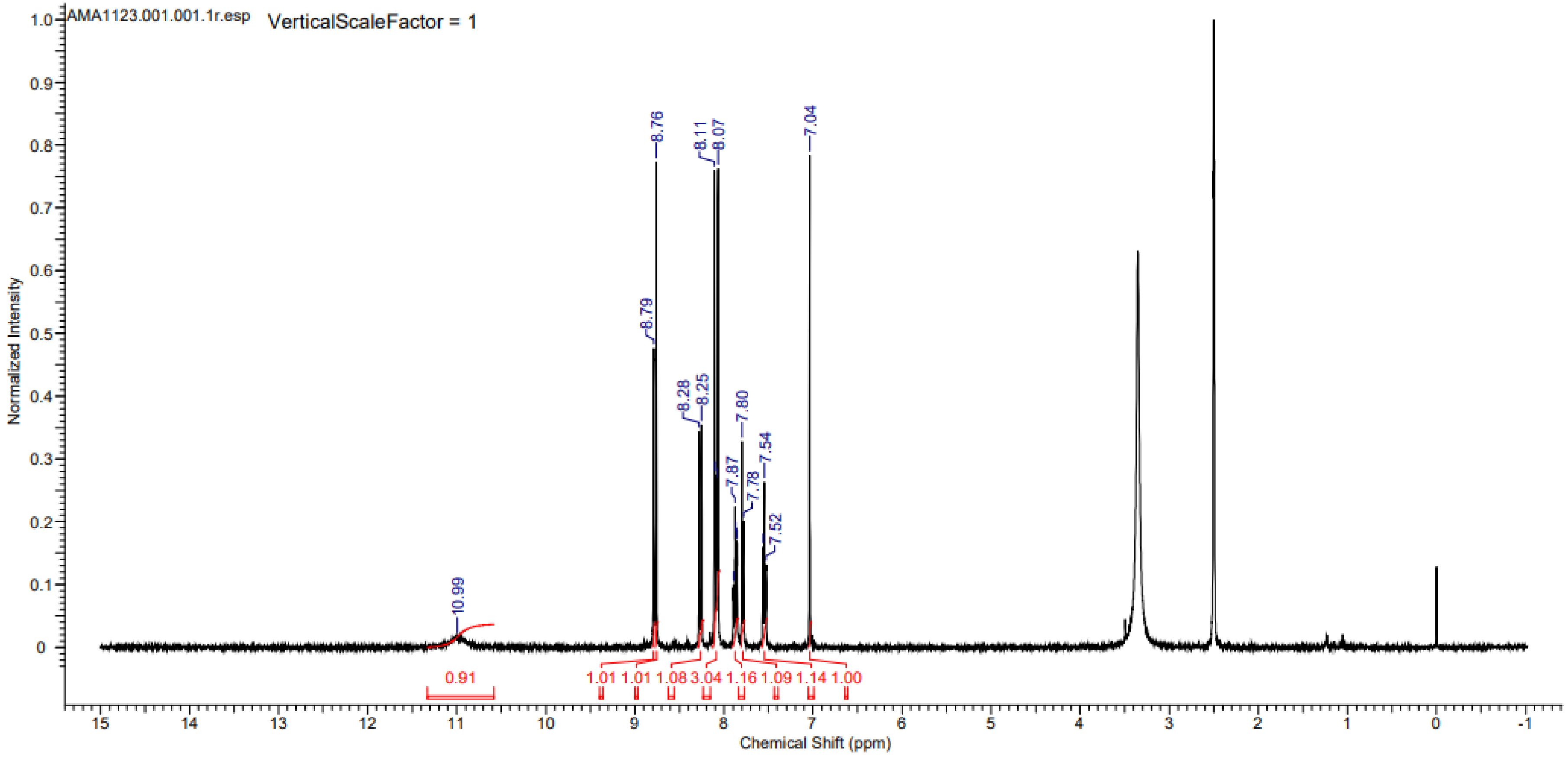

Figure S32. ^{13}C NMR spectrum of compound **22**

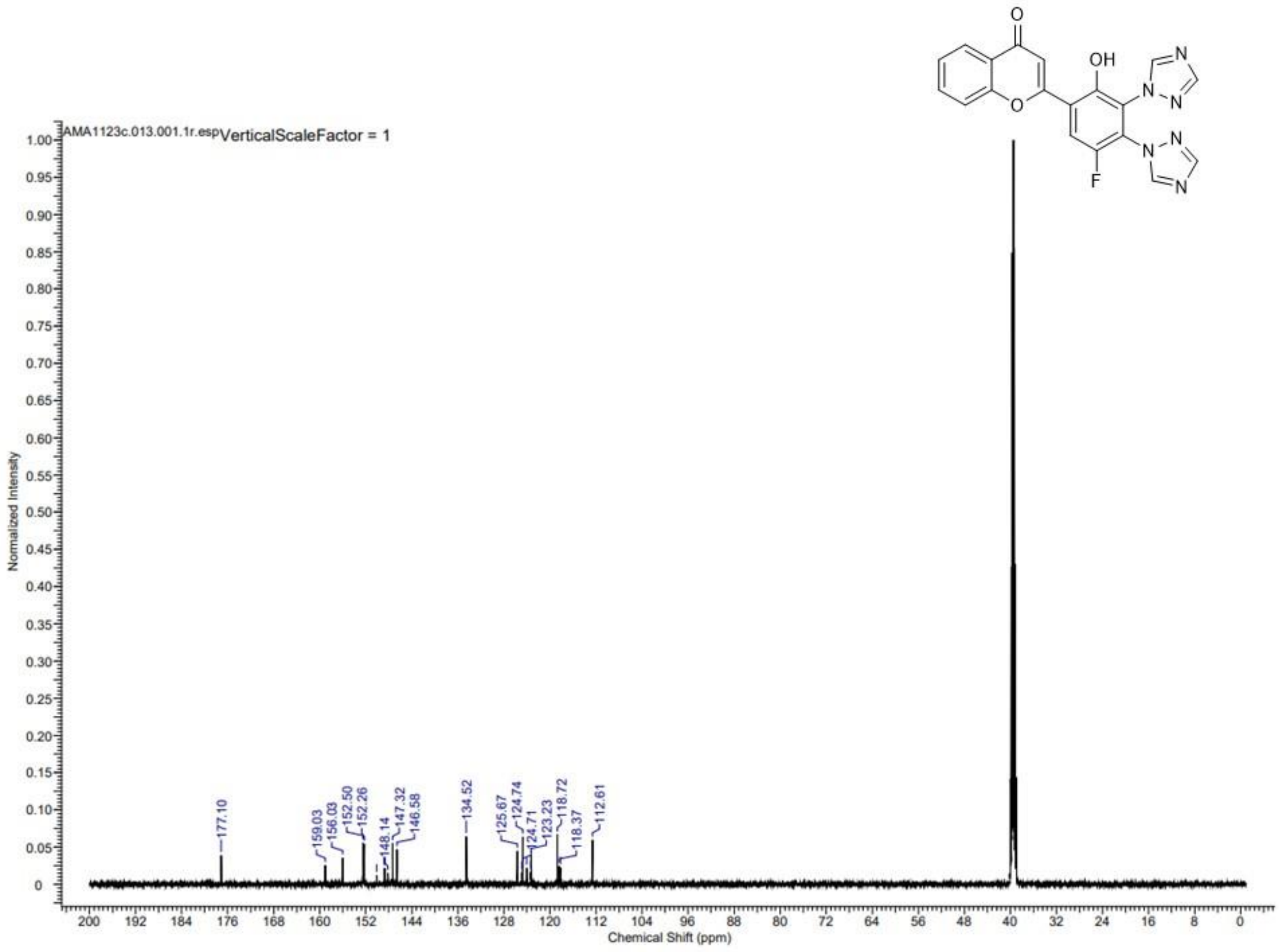

Figure S33. ^{19}F NMR spectrum of compound **22**

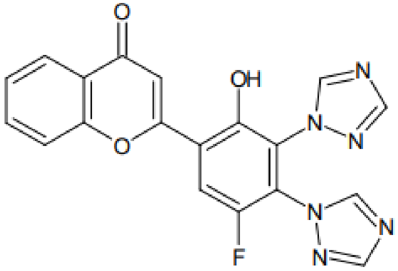

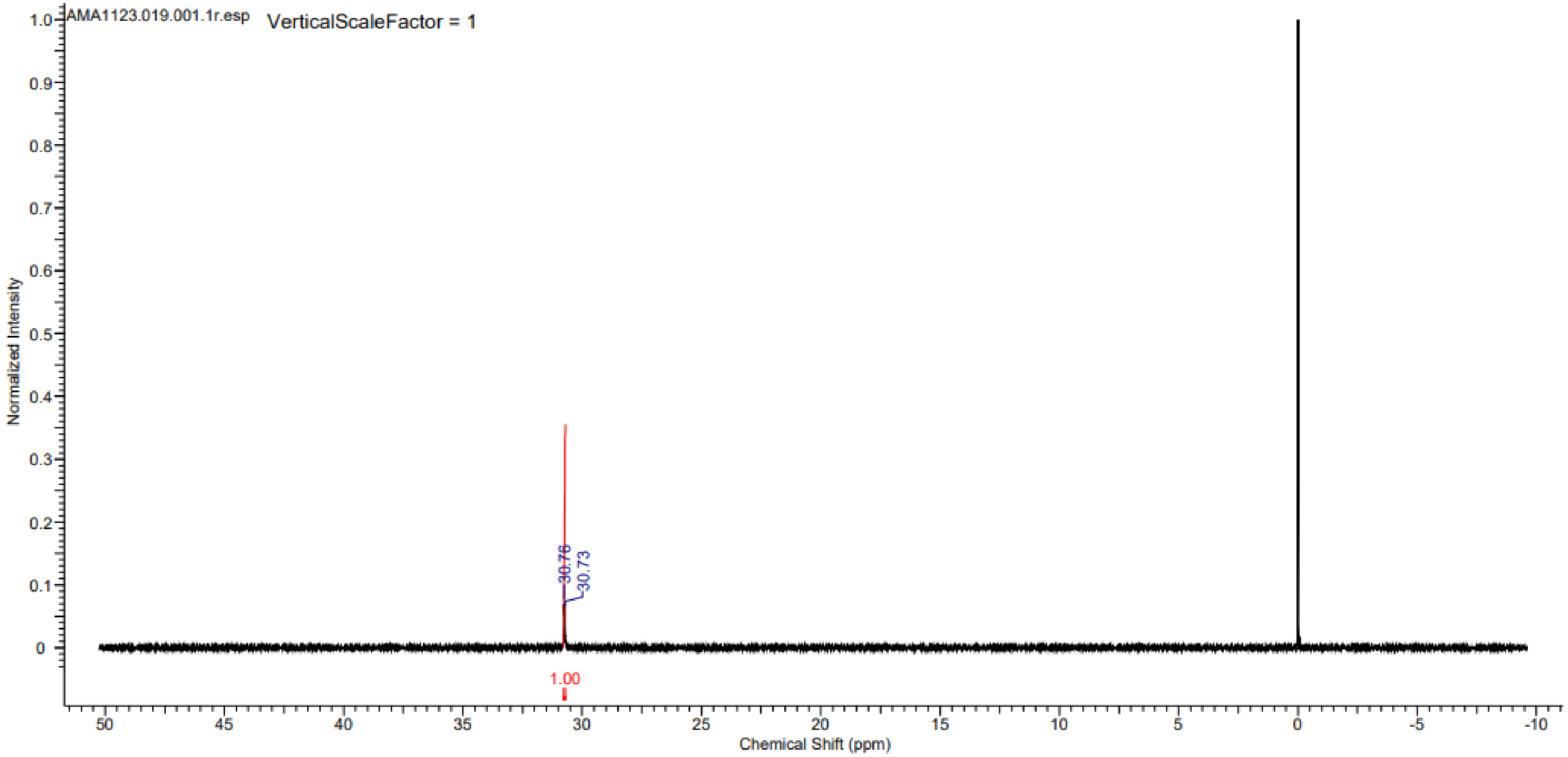

Figure S34. ^{1}H NMR spectrum of compound **23**

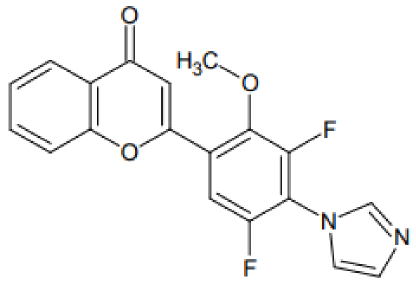

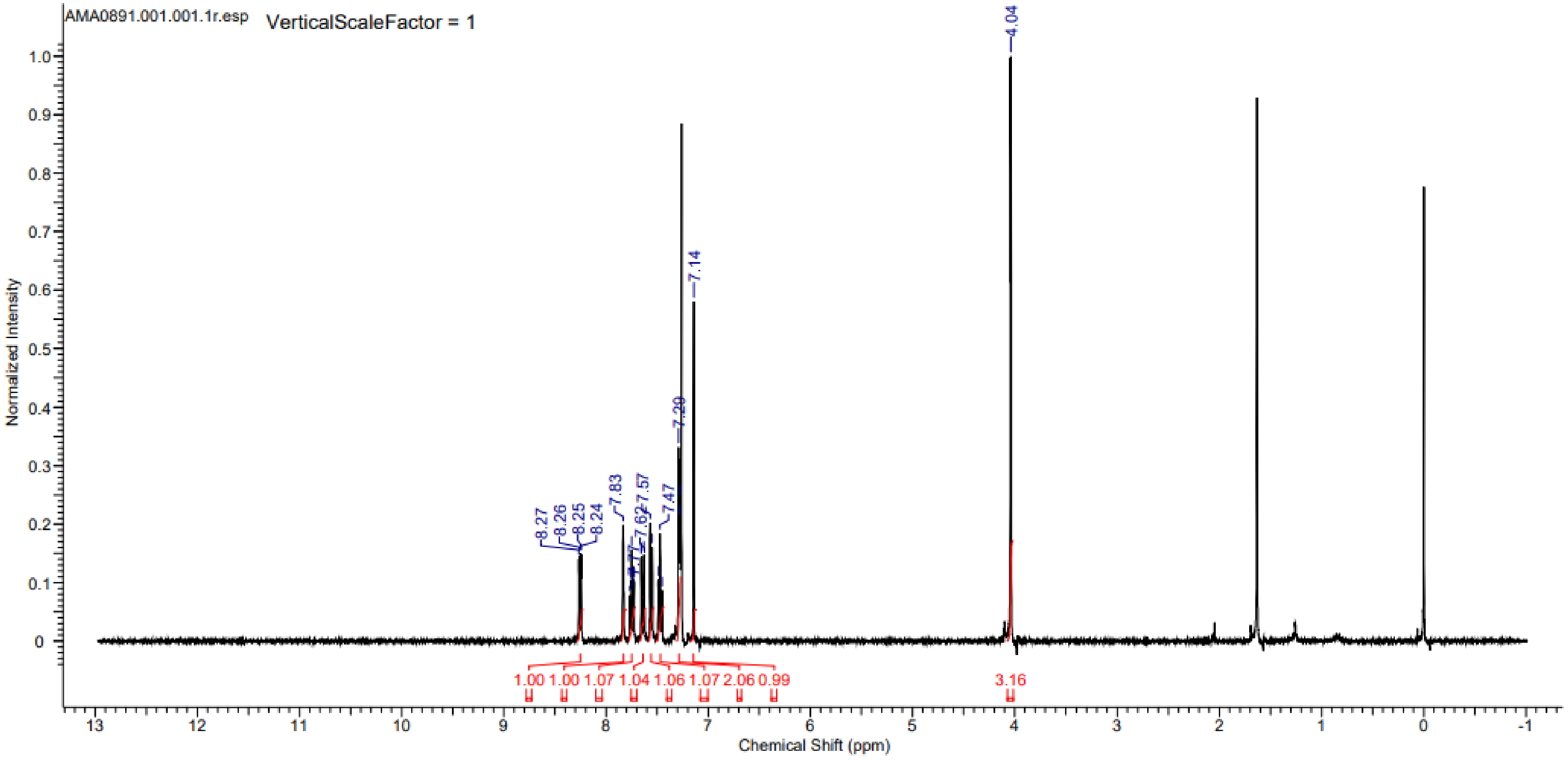

Figure S35. ^{13}C NMR spectrum of compound **23**

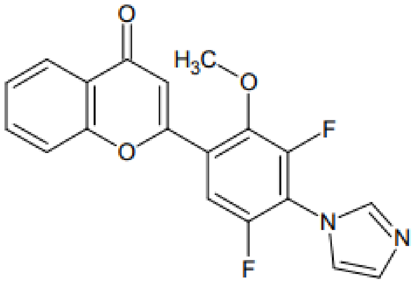

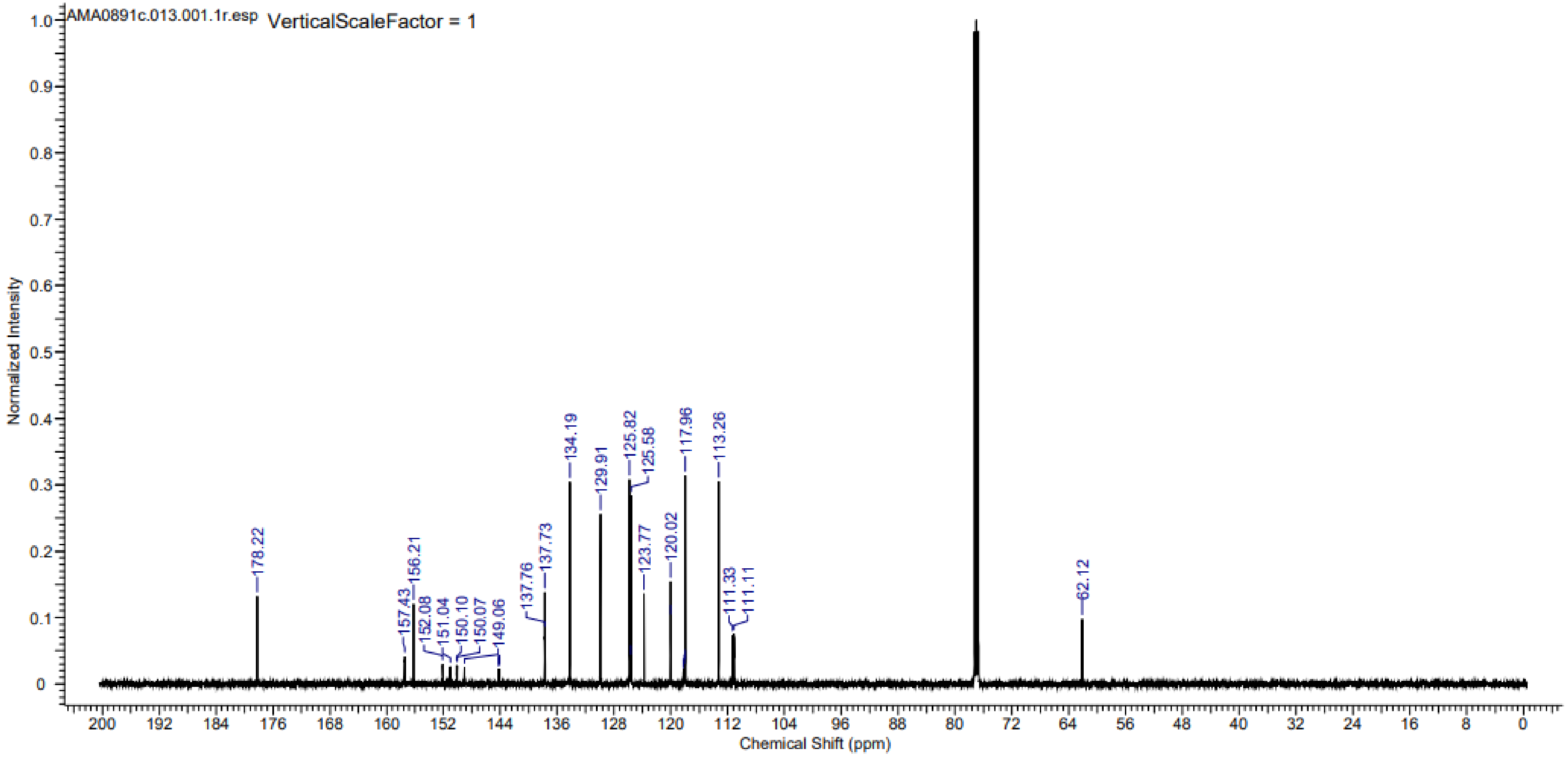

Figure S36. ^{19}F NMR spectrum of compound **23**

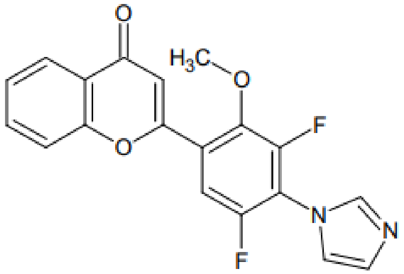

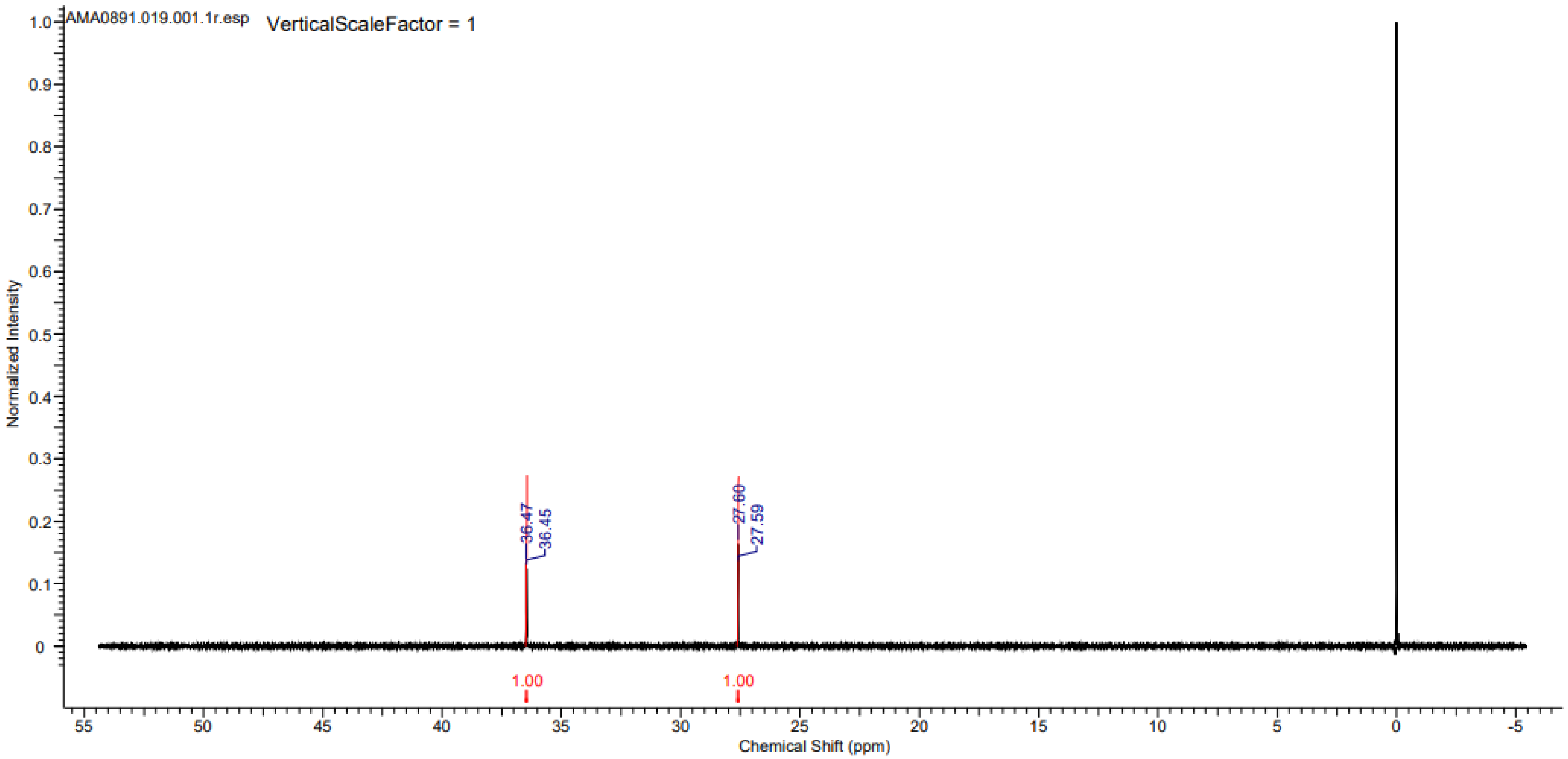

Figure S37. ^{1}H NMR spectrum of compound **26**

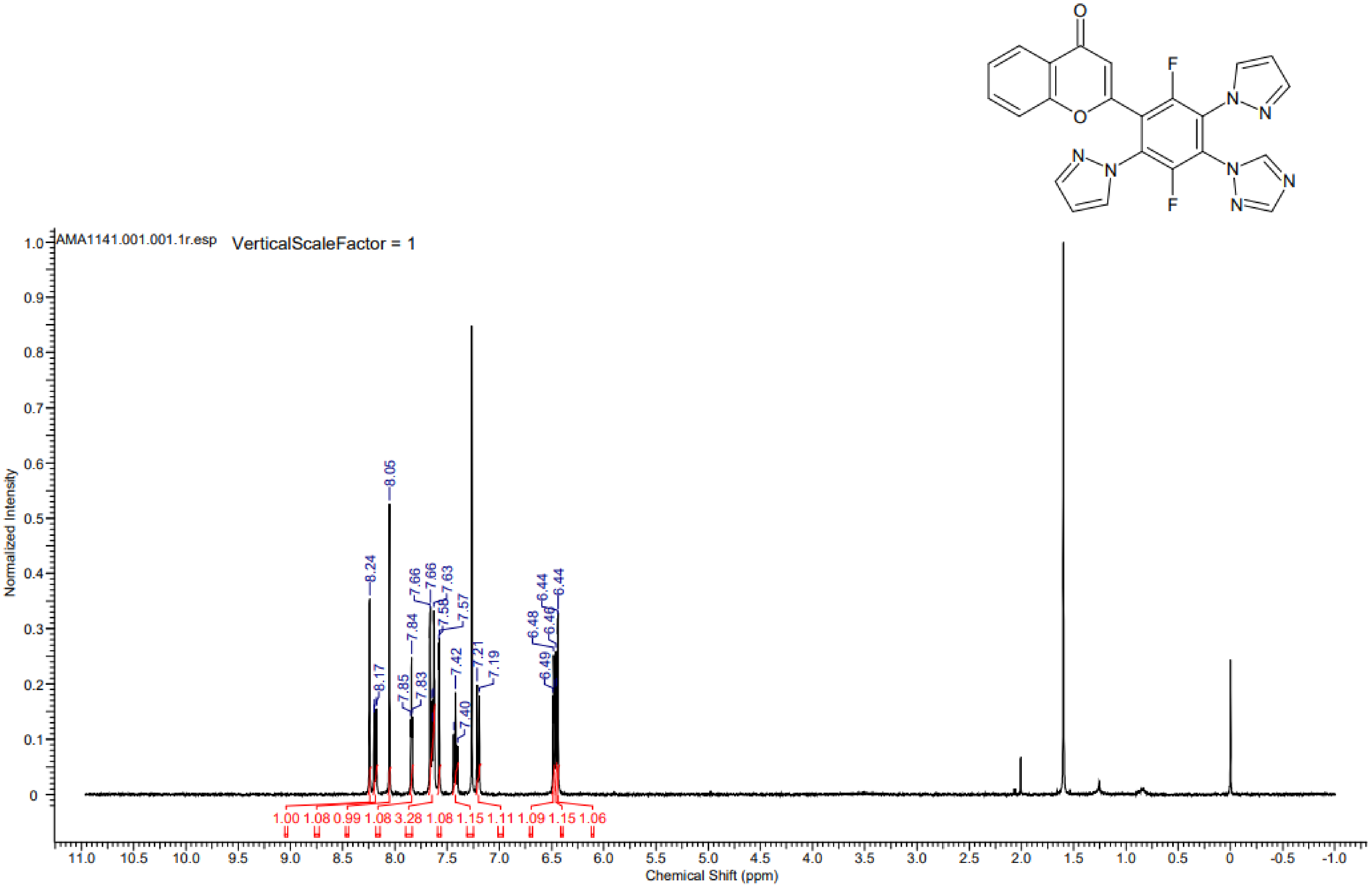

Figure S38. ^{13}C NMR spectrum of compound **26**

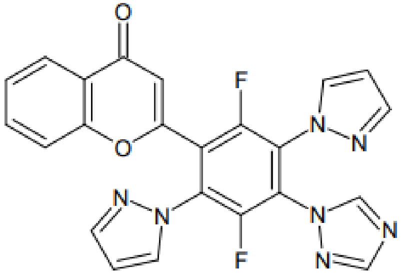

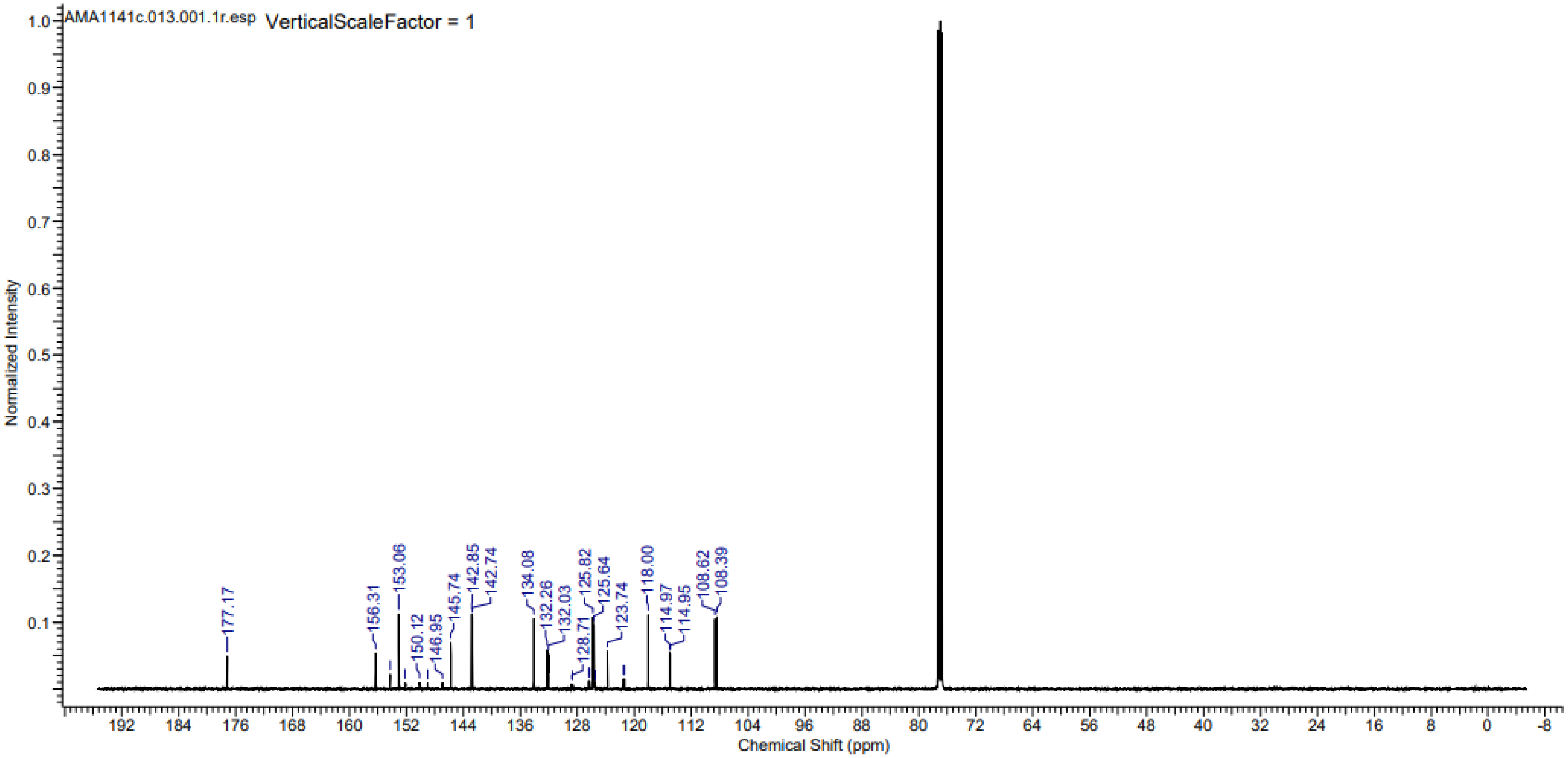

Figure S39. ^{19}F NMR spectrum of compound **26**

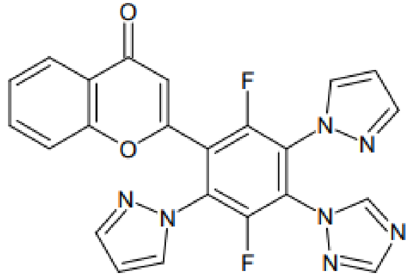

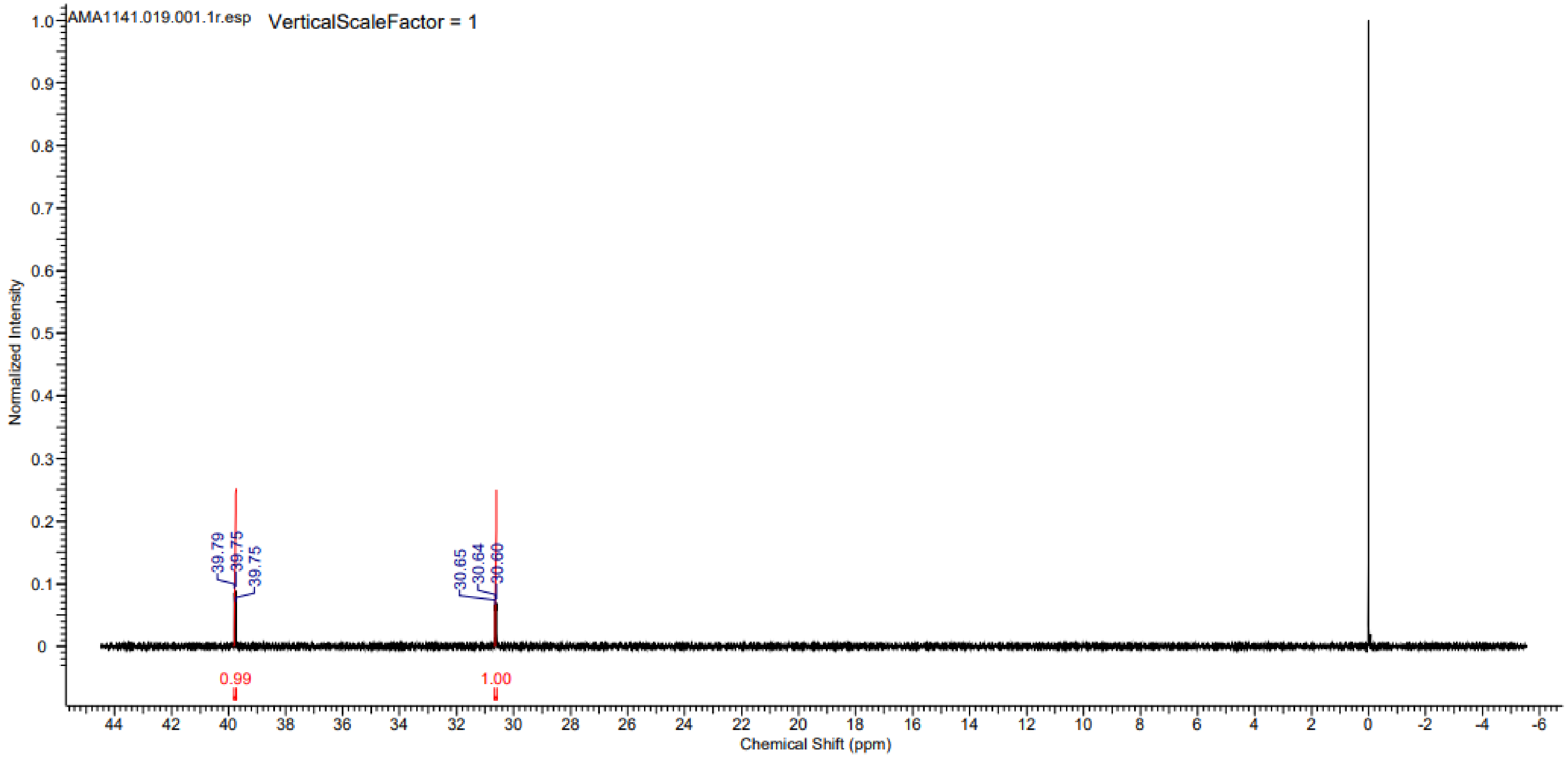

Figure S40. ^{1}H NMR spectrum of compound **27**

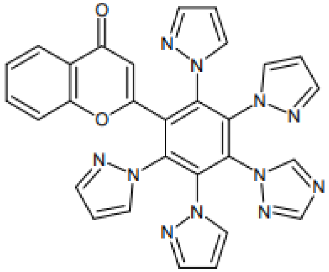

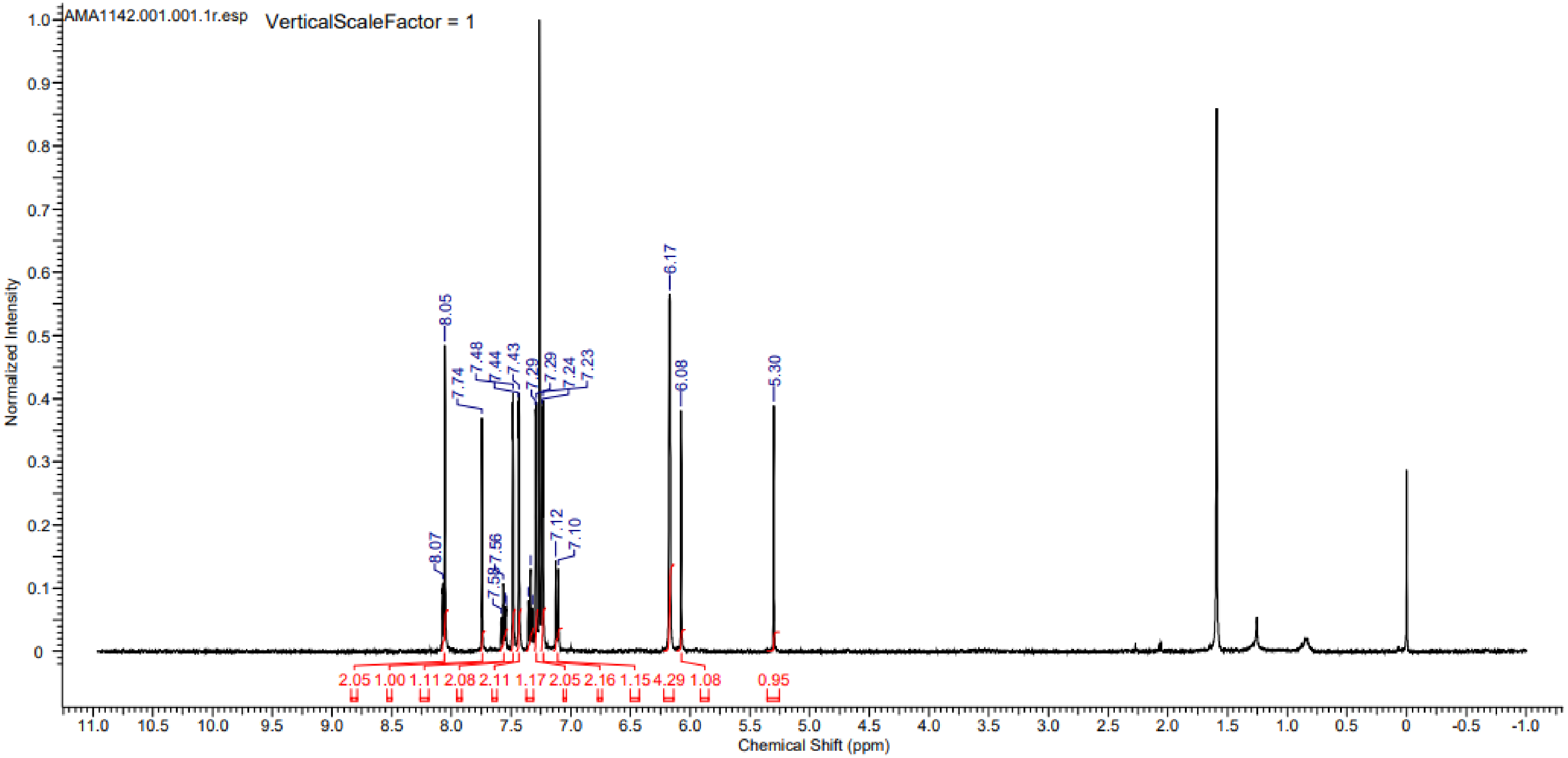

Figure S41. ^{13}C NMR spectrum of compound **27**

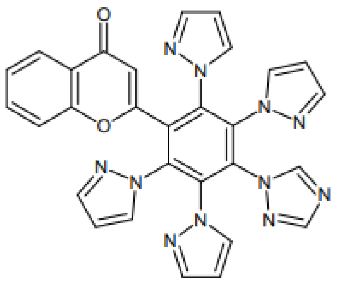

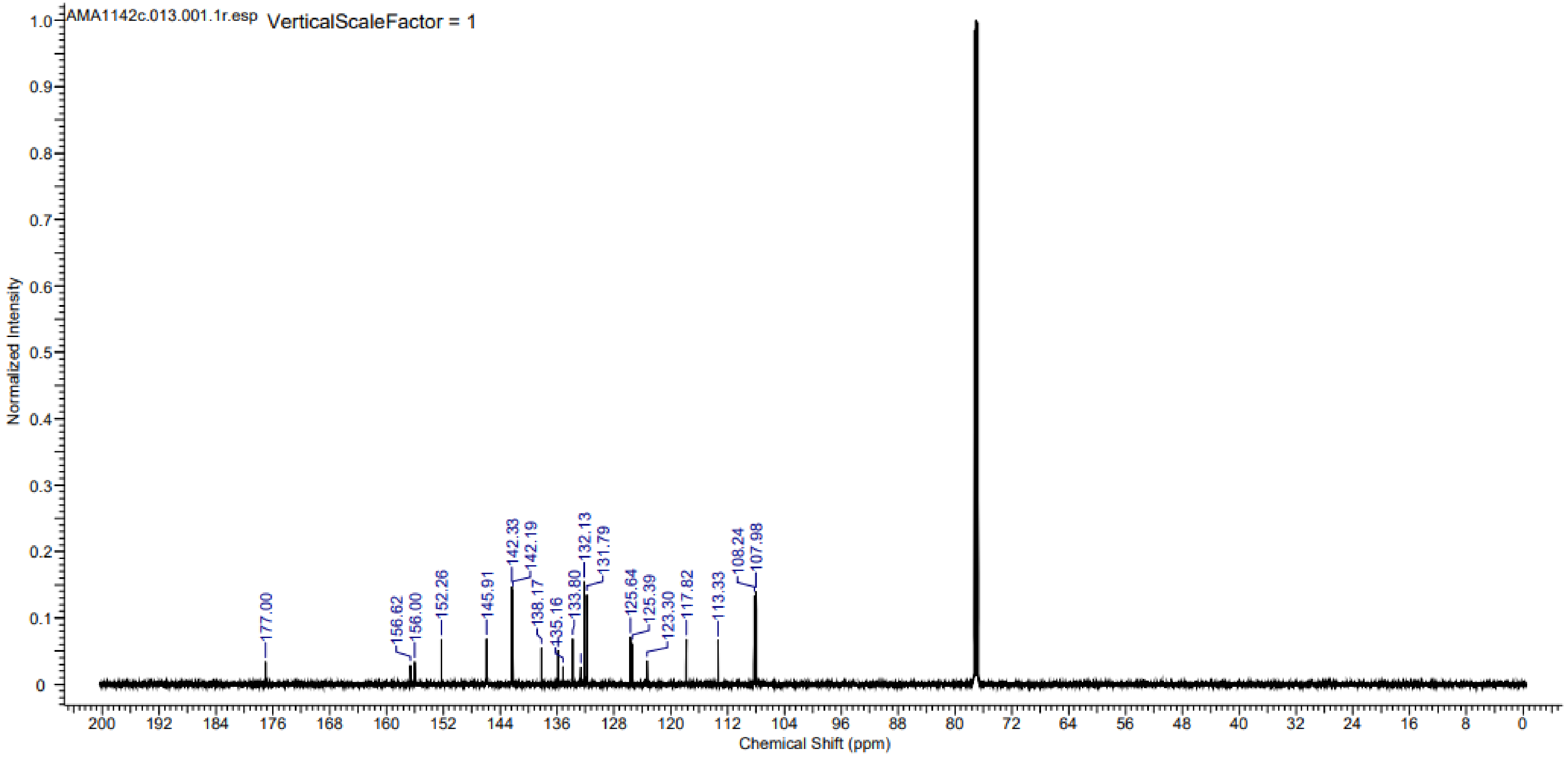

Figure S42. ^{1}H NMR spectrum of compound **28**

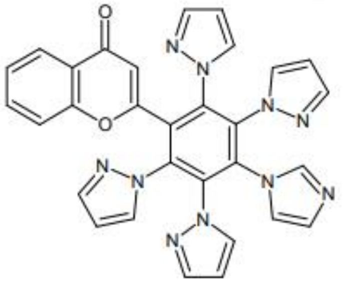

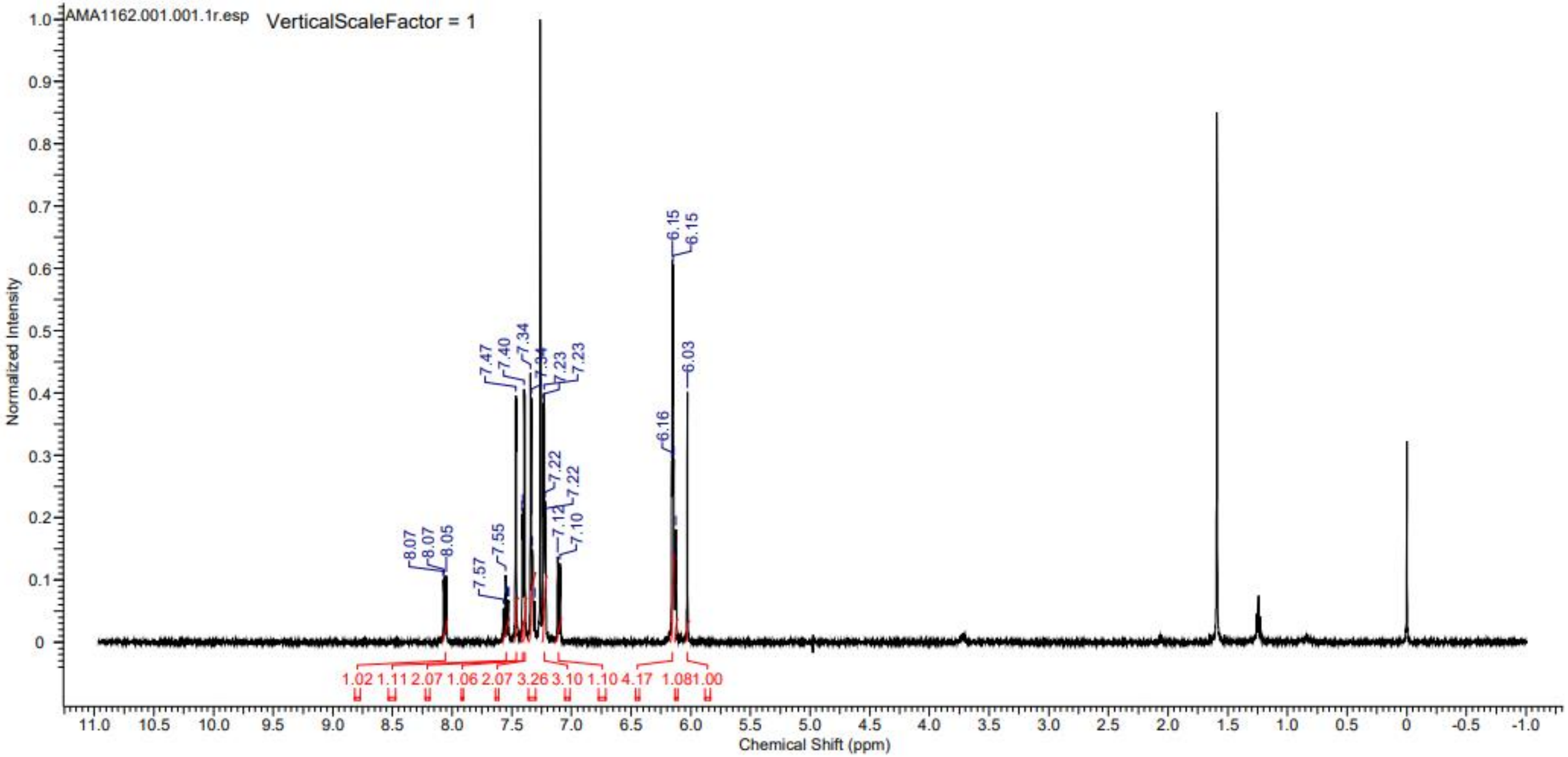

Figure S43. ^{13}C NMR spectrum of compound **28**

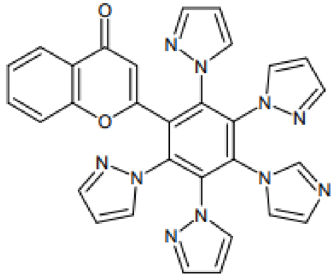

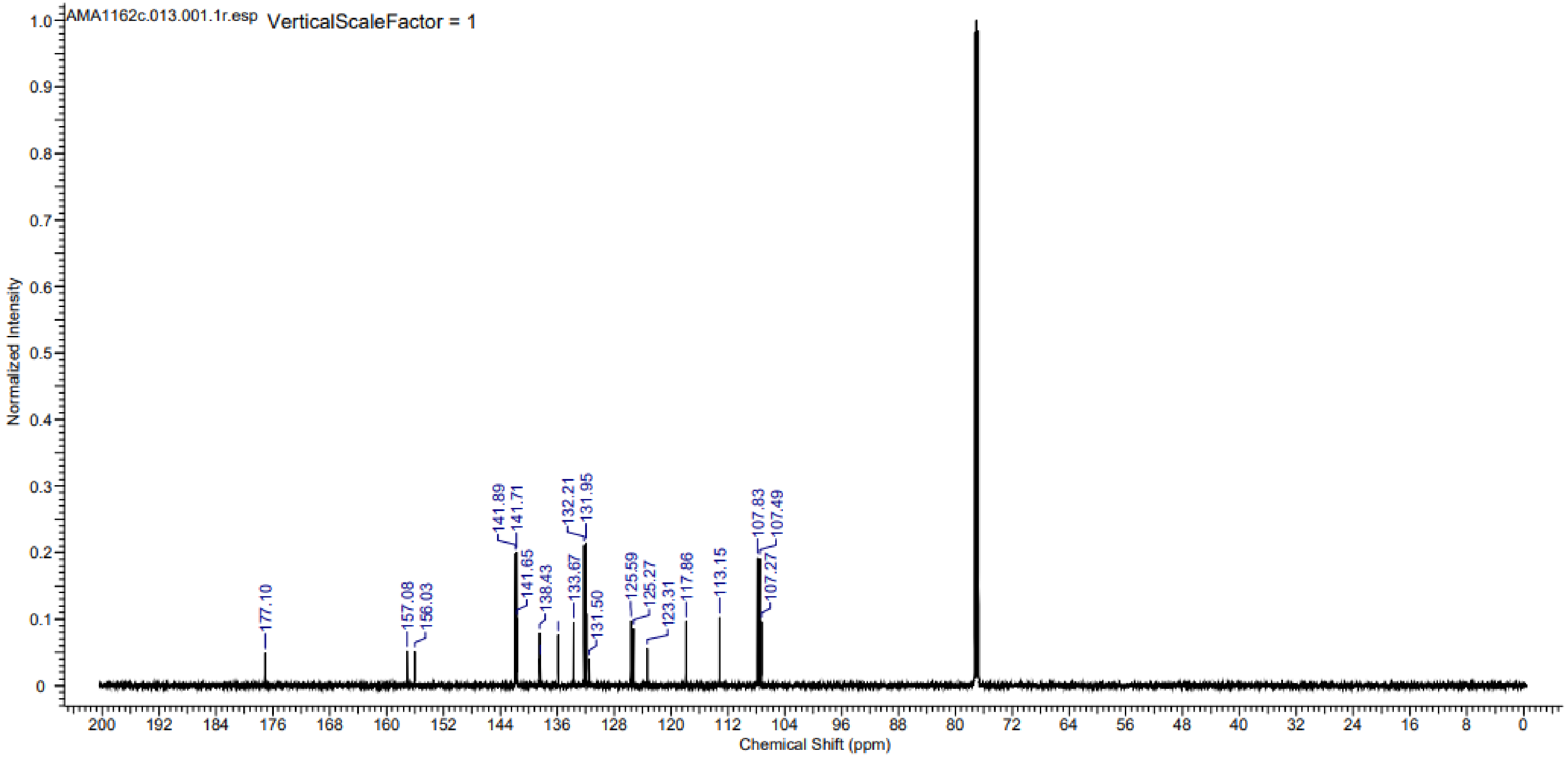

Figure S44. ^{1}H NMR spectrum of compound **29**

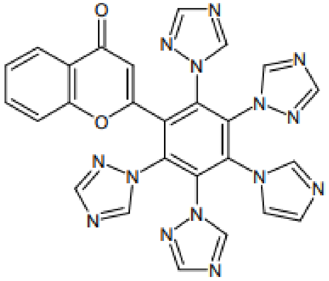

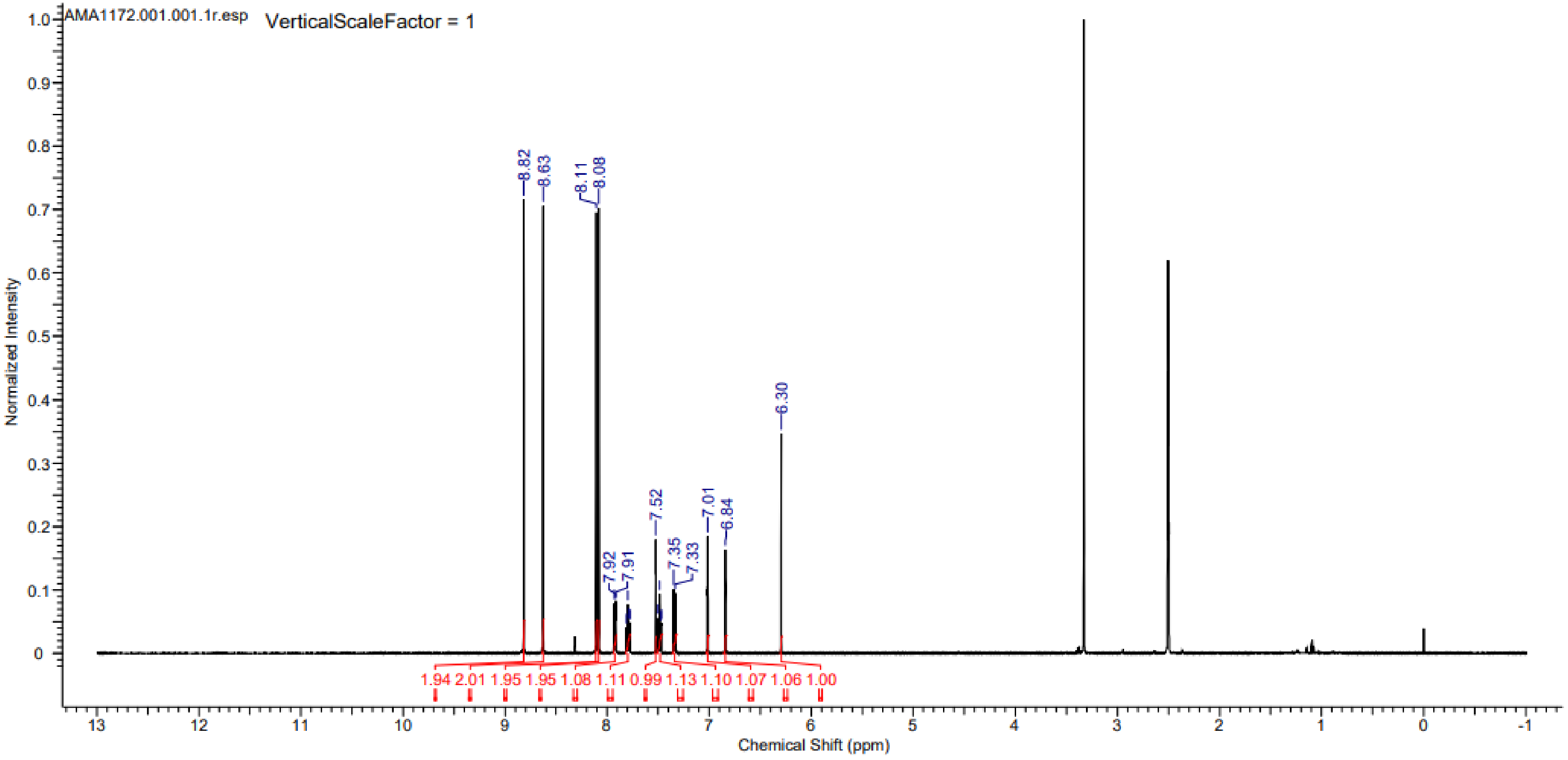

Figure S45. ^{13}C NMR spectrum of compound **29**

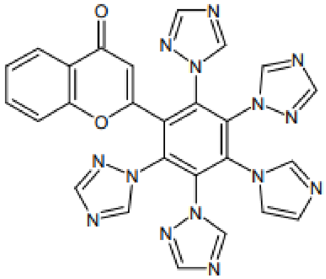

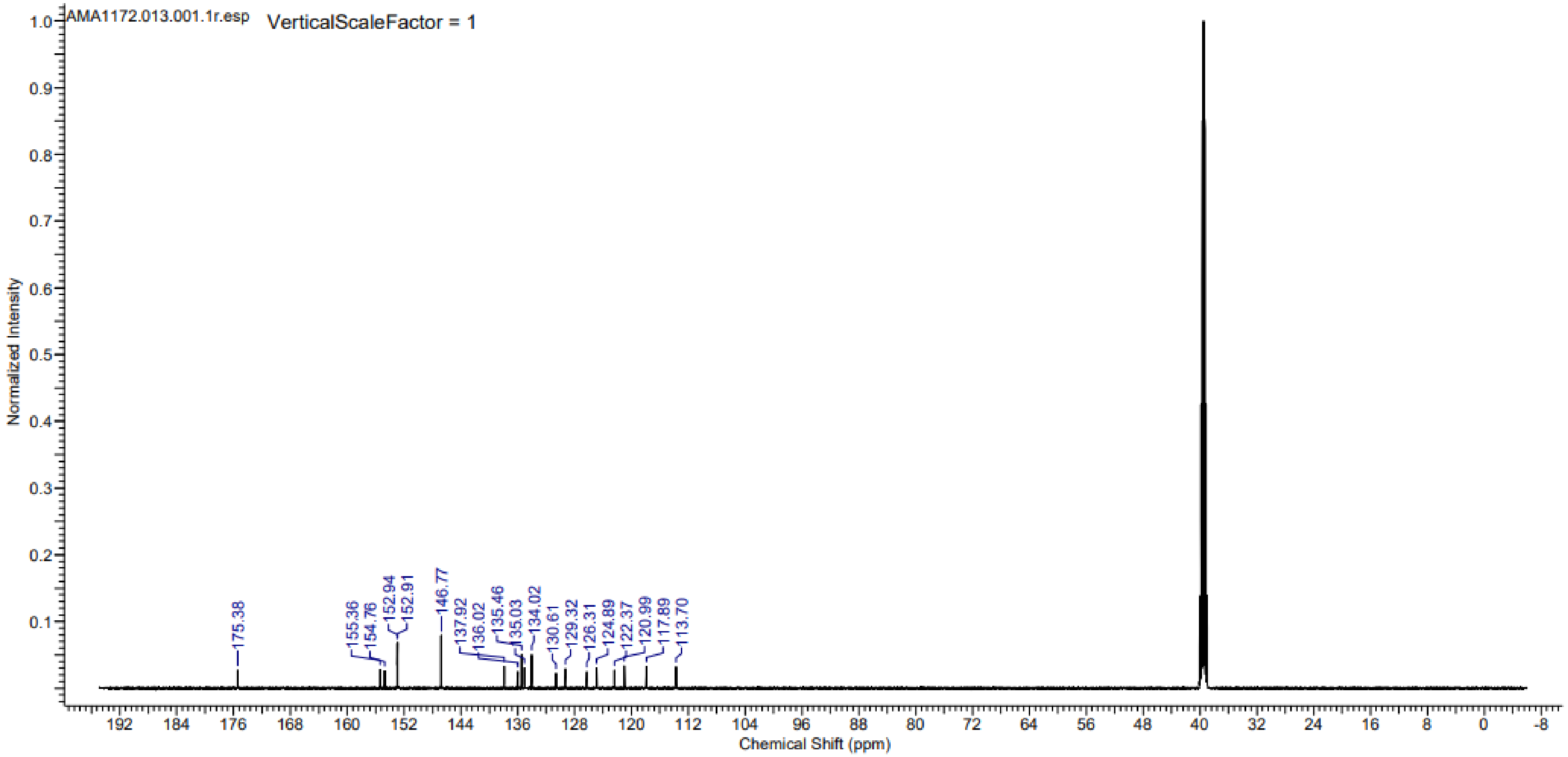

Figure S46. ^{1}H NMR spectrum of compound **31**

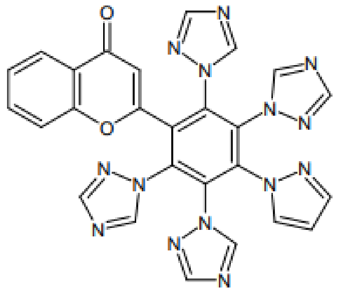

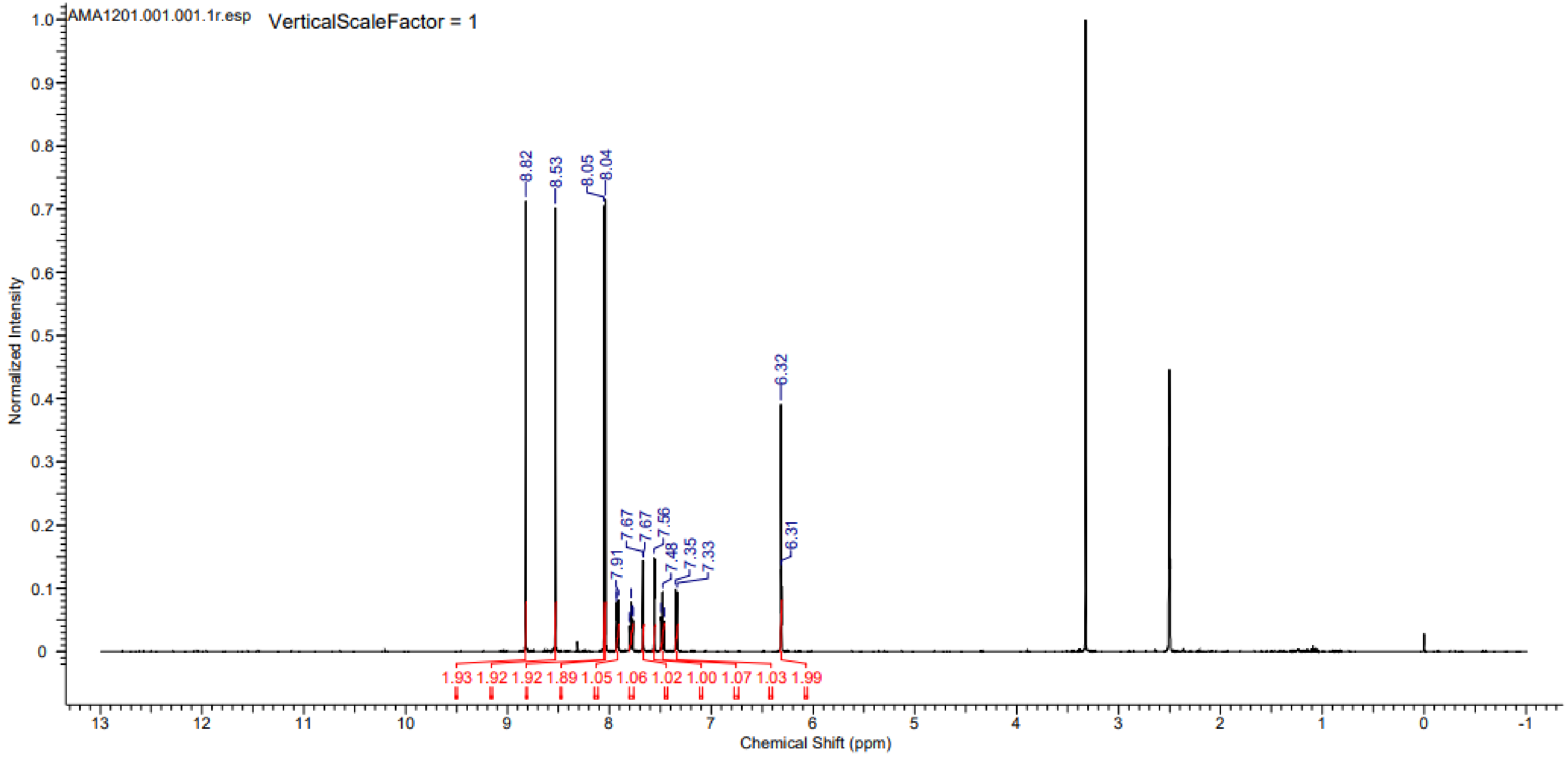

Figure S47. ^{13}C NMR spectrum of compound **31**

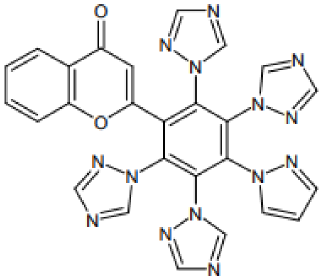

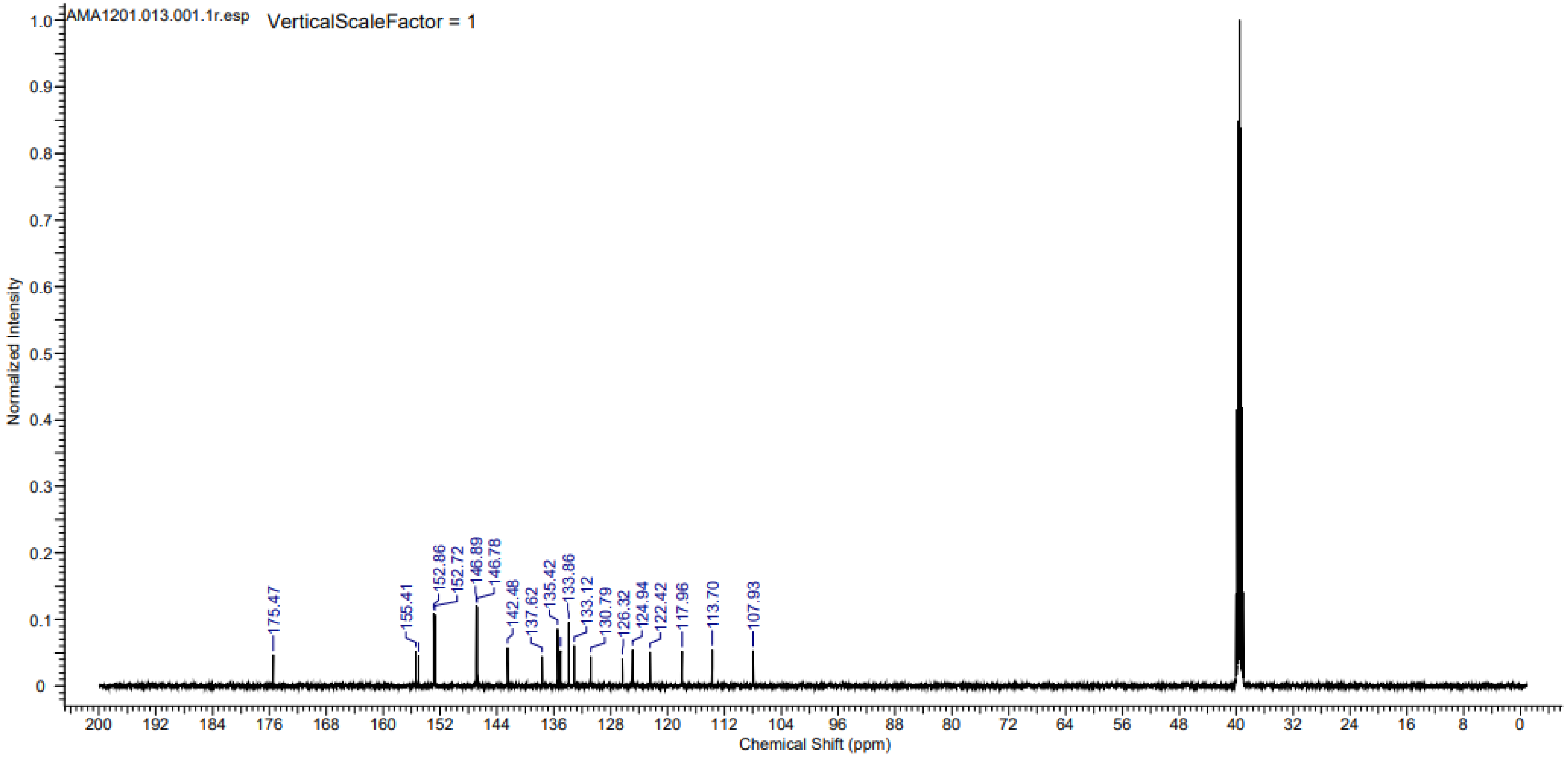

Supplement: Supplementary file 1 [file molecules-28-00869-s001.zip › molecules-2165237-supplementary.pdf]
